# Empfehlungen zur Fachweiterbildung von Pflegefachpersonen der Intensivstation bei der Behandlung des Bauchaortenaneurysmas: Ergebnisse eines modifizierten Delphi-Verfahrens mit Expert:innen

**DOI:** 10.1007/s00104-024-02066-1

**Published:** 2024-03-18

**Authors:** Christian-Alexander Behrendt, Jörg Heckenkamp, Andrea Bergsträßer, Arend Billing, Dittmar Böckler, Arno Bücker, Livia Cotta, Konstantinos P. Donas, Gerd Grözinger, Claus-Dieter Heidecke, Irene Hinterseher, Silvio Horn, Arnold Kaltwasser, Andrea Kiefer, Claudia Kirnich-Müller, Lars Kock, Tilo Kölbel, Martin Czerny, Christian Kralewski, Stephan Kurz, Axel Larena-Avellaneda, Haitham Mutlak, Alexander Oberhuber, Kyriakos Oikonomou, Manfred Pfeiffer, Karin Pfister, Christian Reeps, Andreas Schäfer, Thomas Schmitz-Rixen, Markus Steinbauer, Claudia Steinbauer, Daniel Strupp, Dietmar Stolecki, Matthias Trenner, Christof Veit, Eric Verhoeven, Christian Waydhas, Christian F. Weber, Farzin Adili

**Affiliations:** 1 Deutsches Institut für Gefäßmedizinische Gesundheitsforschung gGmbH, Berlin, Deutschland; 2 Abt. für Allgemeine und Endovaskuläre Gefäßchirurgie, Asklepios Klinik Wandsbek, Asklepios Medical School, Alphonsstr. 14, 22043 Hamburg, Deutschland; 3 Niels-Stensen-Kliniken, Osnabrück, Deutschland; 4 Deutscher Pflegerat e. V., Berlin, Deutschland; 5 Kommission Krankenhausökonomie, Deutsche Gesellschaft für Gefäßchirurgie und Gefäßmedizin e. V., Berlin, Deutschland; 6 Klinik für Gefäßchirurgie und Endovaskuläre Chirurgie, Universitätsklinikum Heidelberg, Heidelberg, Deutschland; 7 Klinik für Diagnostische und Interventionelle Radiologie, Universitätsklinikum des Saarlandes, Homburg, Deutschland; 8 Rhein Main Vascular Center, Klinik für vaskuläre und endovaskuläre Chirurgie, Asklepios Kliniken Langen, Paulinen Wiesbaden und Seligenstadt, Langen, Deutschland; 9 Abt. für Diagnostische und Interventionelle Radiologie, Universitätsklinikum Tübingen, Tübingen, Deutschland; 10 Institut für Qualität und Transparenz im Gesundheitswesen (IQTIG), Berlin, Deutschland; 11 Klinik für Gefäßchirurgie, Universitätsklinikum Ruppin-Brandenburg, Medizinische Hochschule Brandenburg, Neuruppin, Deutschland; 12 Gefäßchirurgie, Alexianer St. Josefs Krankenhaus Potsdam, Potsdam, Deutschland; 13 Sektion Pflegeforschung und Pflegequalität, Deutsche Interdisziplinäre Vereinigung für Intensiv- und Notfallmedizin e. V., Berlin, Deutschland; 14 Deutscher Berufsverband für Pflegeberufe (DBfK) Bundesverband e. V., Berlin, Deutschland; 15 Pflegedirektion, Alexianer St. Josefs Krankenhaus Potsdam, Potsdam, Deutschland; 16 Klinik für Gefäßchirurgie, Immanuel Albertinen Diakonie, Hamburg, Deutschland; 17 Klinik für Gefäßmedizin, Universitätsklinikum Hamburg-Eppendorf, Hamburg, Deutschland; 18 Abteilung für Herz- und Gefäßchirurgie, Universitätsklinikum Freiburg, Freiburg, Deutschland; 19 Medizinische Fakultät, Albert Ludwigs Universität Freiburg, Freiburg, Deutschland; 20 Kompetenz-Centrum Qualitätssicherung (KCQ), Medizinischer Dienst Baden-Württemberg, Tübingen, Deutschland; 21 Klinik für Herz‑, Thorax- und Gefäßchirurgie, Deutsches Herzzentrum der Charité (DHZC), Berlin, Deutschland; 22 Charité – Universitätsmedizin Berlin, Corporate Member of Freie Universität Berlin and Humboldt Universität zu Berlin, Berlin, Deutschland; 23 Abteilung für Gefäß- und endovaskuläre Chirurgie, Asklepios Klinik Altona, Asklepios Medical School, Hamburg, Deutschland; 24 Klinik für Anästhesiologie, Intensiv- und Schmerzmedizin, SANA Klinikum Offenbach, Offenbach, Deutschland; 25 Klinik für Vaskuläre und Endovaskuläre Chirurgie, Uniklinik Münster, Münster, Deutschland; 26 Abteilung für Gefäß- und Endovaskularchirurgie, Universitätsklinikum Frankfurt, Frankfurt, Deutschland; 27 Interessenvertretung Patienten-&-Versicherte, Sörgenloch, Deutschland; 28 Universitäres Gefäßzentrum Ostbayern, Abteilung für Gefäßchirurgie, Universitätsklinikum Regensburg, Regensburg, Deutschland; 29 Bereich Gefäß- und Endovaskuläre Chirurgie, Uniklinikum Dresden, Dresden, Deutschland; 30 Deutsche Gesellschaft für Pflegewissenschaft e. V., Duisburg, Deutschland; 31 Deutsche Gesellschaft für Chirurgie e. V., Berlin, Deutschland; 32 Klinik für Gefäßchirurgie, Gefäßzentrum, Barmherzige Brüder Regensburg, Regensburg, Deutschland; 33 Katholische Akademie für Berufe im Gesundheits- und Sozialwesen, Regensburg, Deutschland; 34 Intensivpflege, Asklepios Klinik Wandsbek, Hamburg, Deutschland; 35 Deutsche Gesellschaft für Fachkrankenpflege und Funktionsdienste e. V., Berlin, Deutschland; 36 St. Josefs Hospital Wiesbaden, Wiesbaden, Deutschland; 37 BQS Institut, Hamburg, Deutschland; 38 Klinikum Nürnberg und Paracelsus Medizinische Privatuniversität, Nürnberg, Deutschland; 39 Deutsche Interdisziplinäre Vereinigung für Intensiv- und Notfallmedizin e. V., Berlin, Deutschland; 40 Klinik Für Unfall‑, Hand- und Wiederherstellungschirurgie, Universitätsklinikum Essen, Universität Duisburg-Essen, Essen, Deutschland; 41 Abteilung für Anästhesiologie, Intensiv- und Notfallmedizin, Asklepios Klinik Wandsbek, Hamburg, Deutschland; 42 Klinik für Anästhesiologie, Intensivmedizin und Schmerztherapie, Universitätsklinik Frankfurt, Frankfurt am Main, Deutschland; 43 Klinik für Gefäßmedizin, Gefäßchirurgie und Endovaskuläre Chirurgie, Klinikum Darmstadt, Darmstadt, Deutschland

**Keywords:** Aorta, Qualifikation, Pflege, Konsensus, Qualitätssicherung, Aorta, Qualification, Care, Consensus, Quality improvement

## Abstract

**Einleitung:**

Die medizinischen Weiterentwicklungen in den vergangenen 15 Jahren und die veränderte Versorgungsrealität bei der flächendeckenden elektiven Behandlung des Bauchaortenaneurysmas machen eine Reevaluation der Qualitätssicherungsrichtlinie des Gemeinsamen Bundesausschuss in Deutschland (QBAA-RL) erforderlich. Diese fordert derzeit in der aktuellen Fassung eine Fachweiterbildungsquote für Pflegefachpersonen der Intensivstation in Höhe von 50 %. Die Quote wurde 2008 auf dem Boden von Expertenmeinungen festgelegt, ohne dass bisher eine direkte empirische Evidenzbasis dafür existiert.

**Methoden:**

Vertreter:innen aus den Bereichen Patient:innenvertreter, Ärzt:innen, Pflegefachpersonen sowie weiteren relevanten Schnittstellenbereichen wurden zur Teilnahme an einem modifizierten Delphi-Verfahren eingeladen. Nach einer umfassenden narrativen Literaturrecherche, einer Umfrage sowie Fokusgruppendiskussionen mit nationalen und internationalen Expert:innen erfolgte die Durchführung von insgesamt drei anonymisierten onlinebasierten Abstimmungsrunden, bei denen zuvor festgelegte Kernaussagen mit einer 4‑Punkt-Likert-Skala („stimme ganz und gar nicht zu“ bis „stimme voll und ganz zu“) bewertet wurden. Das Expert:innenpanel hat außerdem eine Empfehlung für eine Mindestquote für die Fachweiterbildung von Pflegefachpersonen auf der Intensivstation bei der Behandlung des Bauchaortenaneurysmas festgelegt, wobei a priori eine Zustimmung in Höhe von 80 % der Teilnehmenden als Konsensusgrenze festgelegt wurde.

**Ergebnisse:**

Insgesamt haben 37 Expert:innen an den Diskussionen und drei sukzessiven Abstimmungsrunden teilgenommen (Teilnahmerate 89 %). Das Panel hat die Notwendigkeit einer Reevaluation der Richtlinienempfehlungen bestätigt und empfahl die Einführung einer schichtbezogenen Mindestquote in Höhe von 30 % der Vollzeitäquivalente der Pflegefachpersonen der Intensivstation sowie die Einführung strukturierter Förderprogramme zur langfristigen Erhöhung der Quote.

**Schlussfolgerung:**

In diesem nationalen Delphi-Verfahren mit ärztlichen und pflegerischen Expert:innen sowie Patientenvertreter:innen wurde der grundsätzliche Nutzen und Bedarf der beruflichen Fachqualifikation im Bereich der Intensivmedizin bestätigt. Die entsprechenden Mindestquoten für eine Fachweiterbildung von Intensivpflegefachpersonen sollten demnach ohne Einschränkung auf spezifische Leistungsgruppen generell gelten. Das Expert:innenpanel fordert eine schichtbezogene Mindestquote an Intensivpflegefachpersonen mit Fachweiterbildung in Höhe von 30 % der Pflegefachpersonen auf der Intensivstation und die verpflichtende Einführung strukturierter und transparenter Förderprogramme zu deren langfristiger Erhöhung.

**Zusatzmaterial online:**

Zusätzliche Informationen sind in der Online-Version dieses Artikels (10.1007/s00104-024-02066-1) enthalten.

## Abstract (en)

**Introduction:**

The medical development in the previous 15 years and the changes in treatment reality of the comprehensive elective treatment of abdominal aortic aneurysms necessitate a re-evaluation of the quality assurance guidelines of the Federal Joint Committee in Germany (QBAA-RL). In the current version this requires a specialist further training quota for nursing personnel in intensive care wards of 50%. The quota was determined in 2008 based on expert opinions, although a direct empirical evidence base for this does not exist.

**Methods:**

Representatives from the fields of patient representation, physicians, nursing personnel and other relevant interface areas were invited to participate in a modified Delphi procedure. Following a comprehensive narrative literature search, a survey and focus group discussions with national and international experts, a total of three anonymized online-based voting rounds were carried out for which previously determined key statements were assessed with a 4‑point Likert scale (totally disagree up to totally agree). In addition, the expert panel had also defined a recommendation for a minimum quota for the specialist training of nursing personnel on intensive care wards in the treatment of abdominal aortic aneurysms, whereby an a priori agreement of 80% of the participants was defined as the consensus limit.

**Results:**

Overall, 37 experts participated in the discussions and three successive voting rounds (participation rate 89%). The panel confirmed the necessity of a re-evaluation of the guideline recommendations and recommended the introduction of a shift-related minimum quota of 30% of the full-time equivalent of nursing personnel on intensive care wards and the introduction of structured promotional programs for long-term elevation of the quota.

**Conclusion:**

In this national Delphi procedure with medical and nursing experts as well as representatives of patients, the fundamental benefits and needs of professional specialist qualifications in the field of intensive care medicine were confirmed. The corresponding minimum quota for specialist further training of intensive care nursing personnel should generally apply without limitations to specific groups. The expert panel stipulates a shift-related minimum quota for intensive care nursing personnel with specialist training of 30% of the nursing personnel on intensive care wards and the obligatory introduction of structured and transparent promotion programs for the long-term enhancement.

## Hintergrund

Die prophylaktische Ausschaltung des Bauchaortenaneurysmas (BAA) zur Verhinderung einer Aortenruptur stellt eine zentrale Aufgabe der Gefäßchirurgie und weiterer beteiligter Fächer dar. Während mehrere Screeningstudien aus den 1990er-Jahren für das BAA noch eine Prävalenz von 4–8 % bei älteren Männern beschrieben, fiel sie in den folgenden Jahren auf aktuell etwa 1 % ab [8, 11, 32, 43]. Neben dieser epidemiologischen Entwicklung haben sich sowohl die Therapie des BAA als auch die Art der perioperativen Versorgung in den letzten 30 Jahren grundlegend verändert. So wurde das BAA zu Beginn der 2000er-Jahre noch überwiegend durch einen offen-chirurgischen Aortenersatz behandelt, während aktuelle Versorgungsdaten bereits einen Anteil von bis zu 80 % endovaskulärer Behandlungen mit sog. Stentprothesen (endovaskuläre Aortenreparatur, EVAR) ergaben [5, 8, 9, 41]. Dies hat auch einen unmittelbaren Einfluss auf die postprozedurale Behandlung der Patient:innen. So werden nach EVAR, die zunehmend häufig perkutan durchgeführt werden kann, nur noch etwa 60 % der Patient:innen vorübergehend zur Überwachung auf eine Intensiv- oder Monitorstation verlegt. Die endovaskuläre Prozedur gilt nach aktuellen Leitlinien zur Risikostratifizierung heute nicht mehr als ein Hochrisikoeingriff, sondern als Eingriff mit moderatem Risiko [27]. Dieser Unterschied wird auch in Analysen zur Krankenhaussterblichkeit evident, welche 1–2 % nach EVAR und 5–6 % nach offen-chirurgischem Aortenersatz beträgt [5, 8, 9, 41].

Vor dem Hintergrund sinkender Fallzahlen findet bereits seit mehr als 20 Jahren eine kontroverse Diskussion zu möglichen Zusammenhängen zwischen dem Krankenhausfallvolumen und der perioperativen Sterblichkeit statt [13, 14, 17, 18, 20, 22, 23, 29–31, 33, 34, 38–40, 45, 46, 49]. Bis heute ließ sich in den zahlreichen Beobachtungsstudien allerdings kein robuster Zusammenhang bei EVAR nachweisen, während es zunehmend Hinweise auf den Einfluss von Struktur- und Prozessqualitätsparametern beim offen-chirurgischen Verfahren gibt [10, 18, 38–40]. Folgerichtig blieben vereinzelte Forderungen nach entsprechenden Mindestmengen in der 2008 erlassenen Richtlinie des Gemeinsamen Bundesausschuss (G-BA) über Maßnahmen zur Qualitätssicherung für die stationäre Versorgung bei der Indikation Bauchaortenaneurysma (QBAA-RL) seinerzeit noch unberücksichtigt. Stattdessen wurden verschiedene Prozess- und Strukturqualitätsparameter definiert, um die Versorgungsqualität zu verbessern. Neben der ununterbrochenen Vorhaltung der notwendigen allgemeinen, interventionellen, operativen, anästhesiologischen und intensivmedizinischen Infrastruktur in den behandelnden Einrichtungen, wurden explizit und erstmals Vorgaben zur erweiterten Berufsqualifikation des Pflegedienstes auf den Intensivstationen gemacht [7].

Demnach fordert die QBAA-RL eine Fachweiterbildung im Bereich der Intensivpflege und Anästhesie nach den Kriterien der Deutschen Krankenhausgesellschaft (DKG) bei mindestens 50 % der Pflegefachpersonen der Intensivstation und mindestens eine fachweitergebildete Pflegefachperson pro Schicht. Damit stellt diese Richtlinie zum Bauchaortenaneurysma höhere Anforderungen an die pflegerische Fort- und Weiterbildung als die beiden einzigen anderen Richtlinien mit derartigen Empfehlungen zur Fachweiterbildung: So wird für den Bereich Neonatologie eine Mindestquote von 40 % gefordert und für die minimal-invasiven Herzklappeninterventionen 25 %. In weiteren Richtlinien, etwa zur Notfallmedizin oder aufwendigen intensivmedizinischen Komplexbehandlung, sind dagegen keine entsprechenden Anforderungen enthalten [7].

Aufgrund der 2008 auf dem Evidenzniveau von Expert:innenmeinungen festgelegten Mindestquote ohne empirische Datenbasis und der seitdem grundlegend veränderten Versorgungsrealität wurde auf Initiative des Vorstands der Deutschen Gesellschaft für Gefäßchirurgie und Gefäßmedizin e. V. (DGG) im Sommer 2023 eine Reevaluation der erforderlichen Fachweiterbildung von Pflegefachpersonen durch ein modifiziertes Delphi-Verfahren mit berufsgruppenübergreifenden Expert:innen eingeleitet.

Die Kernfrage dieses Konsensusverfahrens war, wie hoch die Mindestquote an Pflegefachpersonen mit einer entsprechenden Fachweiterbildung vor dem Hintergrund der verfügbaren empirischen Daten- bzw. Evidenzbasis oder Expert:innenmeinungen sein sollte. Dabei sollten neben Aspekten der medizinischen Sinnhaftigkeit bzw. Effektivität auch die Umsetzbarkeit und ökonomische Effizienz Berücksichtigung finden.

## Methoden

Es wurde ein modifiziertes Delphi-Verfahren mit Expert:innen aus den folgenden für das Thema relevanten Bereichen durchgeführt: Patient:innenvertreter, Ärzt:innen (Gefäßchirurgie, Radiologie, Anästhesie, Intensivmedizin, Herzchirurgie), Pflegefachpersonen, Qualitätssicherung und Krankenhausökonomie, Medizinischer Dienst, Evaluation medizinischer Leistungen, Krankenhaussektoren (konfessionell, universitär, privat, kommunal; [47]). Die Delphi-Methode wurde in den 1950er-Jahren als strukturiertes mehrstufiges Befragungsverfahren zur Konsensbildung entwickelt und beinhaltet eine moderierte Erfassung anonymisierter Einschätzungen eines Expert:innenpanels [47]. Die Koordinierung, Umsetzung und Moderation des Verfahrens erfolgte durch die medizinisch-wissenschaftliche Leitung des Deutschen Instituts für Gefäßmedizinische Gesundheitsforschung gGmbH (Berlin, Deutschland; [3, 6]).

Die Zusammenstellung des Expert:innenpanels erfolgte auf dem Boden einer Fokusgruppendiskussion im Vorstand der DGG im Juni 2023. Eingeladen wurden die relevanten repräsentativen Gremien, Berufsverbände und Fachgesellschaften sowie klinische Vertreter von Einrichtungen mit einem hohen Fallvolumen in einschlägigen Datenbanken bzw. Krankenhausnavigatoren.

Die Methodik des Delphi-Verfahrens wurde a priori durch ein schriftliches Protokoll festgelegt. Alle eingeladenen Expert:innen und durch die Expert:innen empfohlene Ergänzungen registrierten sich digital für die Teilnahme und legten ihre Interessenkonflikte offen. Den Expert:innen wurde ein Projektexposé zur Verfügung gestellt, das alle notwendigen Hintergrundinformationen enthielt (Electronic Supplement Material).

Das Protokoll sah die Durchführung einer ausführlichen narrativen Literaturrecherche zum Einfluss der Fachweiterbildung von Pflegefachpersonen auf der Intensivstation im Rahmen der Behandlung von Patient:innen mit BAA auf das Behandlungsergebnis vor. Gesucht wurden randomisierte und nichtrandomisierte vergleichende Studien zum Einfluss der Fort- und Weiterbildung nach dem berufsqualifizierenden Berufsabschluss auf die Behandlungsqualität. Hierfür wurden die Suchbegriffe und geeignete Synonyme in deutscher und englischer Sprache zu den Schlagwörtern „Aorta“, „Nursing“, „Staffing“, „Outcomes“, „Mortality“ und „Qualification“ sowie „Training“ über die US-Nationalbibliothek (PubMed) recherchiert. Es fand keine Einschränkung des Veröffentlichungszeitraums statt.

Ergänzend wurden die Mitglieder des VASCUNET-Komitee der European Society for Vascular Surgery (ESVS) und des International Consortium of Vascular Registries (ICVR) sowie alle Expert:innen um die Bereitstellung von Daten, Dokumenten und Literaturempfehlungen zum Thema gebeten [12, 28]. Die internationalen Adressaten des VASCUNET und ICVR, die via E‑Mail befragt wurden (*n* = 35), sollten außerdem beschreiben, ob es in ihrem Land nationale Regulierungen der Fachweiterbildung von Pflegefachpersonen bei der intensivmedizinischen Behandlung des Bauchaortenaneurysmas gibt.

Es fanden insgesamt vier Onlinefokusgruppendiskussionen mit den eingeladenen Expert:innen zur Sammlung von Argumenten und Kernfragen statt.

Auf Basis der gesammelten Kernfragen und Argumente wurden zwei anonyme Abstimmungsrunden in digitaler Form durchgeführt, deren Ergebnisse jeweils anschließend in einer Onlinevideokonferenz besprochen wurden. Alle Expert:innen im Panel wurden via E‑Mail mindestens eine Woche vor der Konferenz eingeladen. Für den Fall, dass die abschließende Empfehlung des Expertenpanels nach zwei Abstimmungsrunden keine Zustimmung von mindestens 80 % der Teilnehmenden erreichte, wurde eine weitere Runde mit einer erzwungenen Zustimmung vs. Ablehnung geplant. Die Protokolle der Expert:innenkonferenzen und Ergebnisse der Abstimmungsrunden wurden allen Teilnehmer:innen unmittelbar im Anschluss per E‑Mail zur Verfügung gestellt.

Die Visualisierung der Ergebnisse erfolgte mit Adobe Illustrator Version 24.1.2 (Adobe, California, USA). Die Erfassung der Abstimmungsergebnisse und deskriptive Analyse erfolgte anonymisiert mit Surveymonkey® (Momentive Europe UC, Ireland). Die Onlinevideokonferenzen erfolgten mit Zoom (San José, Kalifornien, USA).

Die Ergebnisse wurden rein deskriptiv mit absoluten Zahlen (*n*) und Prozenten (%) präsentiert.

## Ergebnisse

### Expert:innenpanel

An dem Delphi-Verfahren beteiligten sich 37 Expert:innen, darunter 6 Frauen (16 %). Alle primär avisierten Bereiche (Patient:innenvertreter, Ärzt:innen, Pflegefachpersonen, Qualitätssicherung und Evaluation) und Krankenhausträger (konfessionell, universitär, privat, kommunal) sowie Versorgungsstufen (Maximal- und Regelversorgung) waren vertreten. Die teilnehmenden ärztlichen Expert:innen vertraten die Fachbereiche Gefäßchirurgie, Anästhesie, Intensivmedizin, Radiologie und Herzchirurgie. Neben 26 Ärzt:innen (70 %) nahmen 8 Pflegefachpersonen (22 %) teil, die 8 Berufs- und Pflegeverbände repräsentierten.

### Literaturrecherche

Bei der narrativen Literaturrecherche konnte ein aktuelles systematisches Cochrane-Review aus dem Jahr 2019 identifiziert werden, dass sich mit verschiedenen Parametern der Pflegequalifikation und assoziierten Behandlungsergebnissen beschäftigt [15]. Die für die Fragestellung relevante Zielgruppe in diesem Review beinhaltete Patient:innen mit Krebs, Asthma, Diabetes, Herzinsuffizienz und chronischen Krankheiten [4, 16, 19, 24–26, 35–37, 42, 44]. Beschrieben wurden dabei Gesundheitssysteme in den USA, UK und Australien. Die Evidenzbasis wurde in allen Bereichen als sehr niedrig oder niedrig bewertet und der Einfluss auf die kurzfristige Sterblichkeit wurde als gering oder nicht vorhanden angesehen. In der Schlussfolgerung kamen die Autor:innen zu dem Ergebnis, dass die wissenschaftliche Basis sehr limitiert sei und dass die Ergebnisse zurückhaltend zu bewerten seien.

Auf dem Boden der Empfehlungen des Expert:innenpanels wurden zwei weitere Beobachtungsstudien identifiziert, die sich mit der Qualifikation von Pflegefachpersonen und dem Behandlungsergebnis beschäftigen [1, 2].

Durch die Expert:innen wurde eine Veröffentlichung mit Strukturvorgaben der Deutschen Interdisziplinären Vereinigung für Intensiv- und Notfallmedizin e. V. (DIVI) genannt, die von Leistungsbereichen unabhängige Empfehlungen zur Fachweiterbildung von Pflegefachpersonen in allen Stufen der Intensivmedizin (I–III) gibt [48]. Originaldaten zur Beantwortung der Fragestellung beinhaltet diese Veröffentlichung nicht und die Untersuchung der referenzierten Arbeiten war ergebnislos.

### Umfrage unter internationalen Expert:innen des VASCUNET und ICVR

Insgesamt sind 35 Expert:innen des VASCUNET und ICVR angeschrieben worden. Spezifische internationale Literatur oder Leitlinienempfehlungen zur Fragestellung waren den Adressaten nicht bekannt. Bis zum Abschluss des Verfahrens haben insgesamt 3 Expert:innen berichtet, dass entsprechende Mindestquoten gelten würden (England, Neuseeland, Schweiz). In England (10,5 Intensivbetten pro 100.000 Einwohner) existiert eine Empfehlung der Faculty of Intensive Care Medicine gemeinsam mit der Intensive Care Society, wonach in Level-2- und Level-3(Critical Care)-Intensivstationen mindestens 50 % der Pflegefachpersonen einen berufserweiternden akademischen Abschluss in Critical Care Nursing besitzen müssen. In Neuseeland (3,6 Intensivbetten pro 100.000 Einwohner) wurden durch den zuständigen Pflegeverband Standards erlassen, wonach mindestens 50 % (und optimalerweise 75 %) der Pflegefachpersonen qualifizierte Critical Care Nurses sein müssen. Im Falle einer Nichterreichung der 50 %-Quote werden zusätzliche personelle Unterstützungen und Förderprogramme gefordert. In der Schweiz (11,8 Intensivbetten pro 100.000 Einwohner) wurde durch die Zertifizierungskommission Intensivstation der Schweizerischen Gesellschaft für Intensivmedizin verpflichtende Kriterien erlassen. Demnach muss mindestens ein Drittel der verlangten, minimalen Vollzeitstellenprozente des Pflegefachpersonals und eine am Bett tätige Pflegefachperson pro Schicht über das Diplom „Experte in Intensivpflege NDS HF“ oder eine gleichwertige Ausbildung verfügen.

### Vorbereitende Fokusgruppendiskussion

Bei der Fokusgruppendiskussion wurden die Methodik sowie der für das Delphi-Verfahren relevante Inhalt der Qualitätssicherungsrichtlinie des G‑BA und das Ergebnis der narrativen Literaturrecherche vorgestellt. Die Teilnehmenden brachten im Anschluss verschiedene Argumente vor, die für die Bewertung der zentralen Fragestellung von potenzieller Relevanz waren (Tab. 1).

**Table 1:** 

Es existiert ein indirekter Evidenzkörper aus Beobachtungsstudien, der einen besseren „Skill-Mix“ des Pflegeteams mit besseren Behandlungsergebnissen assoziiert
Die notwendigen Rechenregeln und die Grundgesamtheit zur Bestimmung einer schichtgenauen Fachkraftquote für die Pflege müssen dezidiert und prüfbar definiert werden
Die Krankenhäuser hatten seit der Einführung der QBAA-RL (2008) Zeit, entsprechende Quoten zu erfüllen. Zum Zeitpunkt des Delphi-Verfahrens gilt die Erreichung der 50 %-Quote nach Ansicht der DIVI als nicht realistisch und zeitnah umsetzbar. Eine nicht repräsentative Umfrage unter DGG-zertifizierten Gefäßzentren ergab eine negative Prüfquote von ca. 30–40 %, während die Evaluation der Richtlinie durch die BQS ergeben hatte, dass ca. 80 % der Krankenhäuser keine Probleme mit der Erfüllung der Vorgaben berichteten
Für eine Änderung bzw. Anpassung der Vorgaben der Richtlinie ist eine entsprechende Evidenzbasis erforderlich, wobei auch ein Expert:innenkonsens als Evidenz gilt
Kleinere Krankenhäuser mit geringerer Personalfluktuation können die 50 %-Quote möglicherweise besser erfüllen als Maximalversorger mit sehr komplexer Intensivmedizin
Durch den Wegfall von Krankenhäusern aus der flächendeckenden Versorgung des Bauchaortenaneurysmas können eine Unterversorgung und Kollateralschaden entstehen
Logistische, administrative und ökonomische Aspekte bei der Personalbeschaffung sollten erst sekundär eine Rolle spielen, da es hier primär um die Patientensicherheit geht
Die Rolle der kurzfristigen Krankenhaussterblichkeit als einziger Indikator der Ergebnisqualität bei der elektiven Versorgung des Bauchaortenaneurysmas ist grundsätzlich fragwürdig
Die Sicherstellung der Qualität der Intensivpflege kann auch über die pro Schicht erforderliche fachlich verantwortliche Pflegefachperson mit entsprechender Fachqualifikation ohne Quote sichergestellt werden. Jedoch erscheint dabei ein schichtbezogener Faktor, der auch die Größe der Intensivstation berücksichtigt, sachgerecht (z. B. prozentual schichtbezogene Fachkraftquote pro potenziell betreibbarem Intensivbett)
Die Qualitätssicherungsrichtlinie simplifiziert die Versorgungsrealität, da eine intensivmedizinische Behandlung nach EVAR nicht zwingend erforderlich ist, während komplexe endovaskuläre Behandlungen der thorakoabdominellen Aorta mit fenestrierten Prothesen nicht reguliert werden
Bei der Einführung und Umsetzung von Quoten gilt auch das Gebot der Umsetzbarkeit und Ökonomie
Eine Expert:innenmeinung aus der Zeit vor 2008 kann auf die nach mehr als 15 Jahren veränderte Versorgungsrealität nicht unmittelbar übertragen werden. Der Anteil minimal-invasiver perkutaner Behandlungen mittels EVAR, die verwendeten Medizinprodukte und die medizinische Behandlung haben sich insgesamt verändert
Das gleichberechtigte Ersatzkonstrukt der „mindestens 5‑jährigen Erfahrung in der Intensivpflege“ wurde nicht ausreichend definiert (z. B. Art, Ort und Umfang der Tätigkeit). Eine Gleichberechtigung unterminiert die Motivation für Pflegefachpersonen, einen qualifizierenden Abschluss zu machen
Aufgrund des Komorbiditäts- und Risikoprofils der Patient:innen mit Behandlung des Bauchaortenaneurysmas gegenüber anderen Risikogruppen (z. B. koronar-interventionell behandelte Patient:innen) ist eine spezifische/höhere Mindestquote für diesen Leistungsbereich nicht erklärbar. Alle Empfehlungen sollten grundsätzlich unabhängig von Leistungsgruppen, Prozeduren oder Krankheitsbildern für alle intensivmedizinischen Leistungen der entsprechenden Stufen gelten
Grundsätzlich sollte eine möglichst breite Fort- und Weiterbildung aller beteiligten Berufsgruppen gefördert werden
Eine Weiterverlegung hin zu Einrichtungen mit höherem Versorgungsniveau ist beim Auftreten von Komplikationen (prä- oder postoperativ) möglich und wird gelebt
Bei der Diskussion erlangt die Mindestqualifikation der Pflegefachpersonen ein Übergewicht gegenüber der Arbeit als Team aus Ärzt:innen, Therapeut:innen und Pfleger:innen. Die Diagnostik und Behandlung der häufigsten Komplikationen, z. B. Pneumonie und Niereninsuffizienz, sind nicht überwiegend von einer Berufsgruppe zu verantworten
Der Fachkräftemangel führt zu einer Regulation des Versorgungsgeschehens
Die Umsetzbarkeit von Personalanforderungen unterscheidet sich international in Abhängigkeit von der Anzahl der Kliniken und Intensivbetten
Wenn ein 100 %-Facharztstandard gefordert wird, sollte auch ein 100 %-Fachpflegeweiterbildungsstandard gefordert werden
Es ist davon auszugehen, dass sich eine Quote in Höhe von 50 % innerhalb der nächsten 5 Jahre nicht durch Förderprogramme erreichen lässt, weshalb umfassende durch Politik und Regulierung sowie Kostenträger zu finanzierende Maßnahmen erforderlich sind
Die Entwicklung im Gesundheitswesen hat zu einer Reduktion der Anzahl von Weiterbildungsstätten/Krankenhäusern geführt, weshalb die Erreichbarkeit geeigneter Einrichtungen auch für die Patient:innen abgenommen hat. Gleichzeitig nehmen damit in Zeiten des Pflegenotstands auch Zugangsmöglichkeiten zu und Umsetzbarkeit für oftmals berufsbegleitende Weiterbildungsmaßnahmen in der Pflege ab

### Delphi-Runde 1

**Figure 1:**
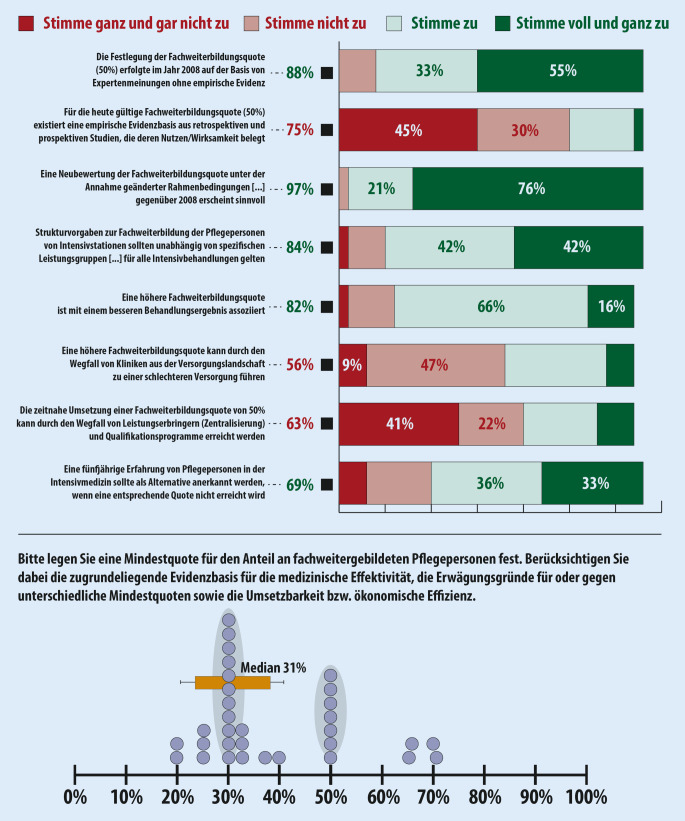

Zur Teilnahme an der ersten Delphi-Abstimmungsrunde wurden 36 Expert:innen eingeladen (Teilnahmequote 89 %, *n* = 32). Insgesamt wurden 8 Statements durch das Expert:innenpanel bewertet, wobei 4 Statements mit über 80 % Zustimmung bewertet wurden (Abb. 1):

- Die Festlegung der Fachweiterbildungsquote (50 %) erfolgte im Jahr 2008 auf der Basis von Expert:innenmeinungen ohne empirische Evidenz. (Zustimmung: 88 %)
- Eine Neubewertung der Fachweiterbildungsquote unter der Annahme geänderter Rahmenbedingungen […] erscheint sinnvoll. (Zustimmung: 97 %)
- Strukturvorgaben zur Fachweiterbildung der Pflegefachpersonen von Intensivstationen sollten unabhängig von spezifischen Leistungsgruppen […] für alle Intensivbehandlungen gelten. (Zustimmung: 84 %)
- Eine höhere Fachweiterbildungsquote ist mit einem besseren Behandlungsergebnis assoziiert. (Zustimmung: 82 %)

Auf die Frage, welche Mindestquote für den Anteil an fachweitergebildeten Pflegefachpersonen festgelegt werden sollte, ergaben sich zwei Abstimmungscluster (30 und 50 %). Der Median der Abstimmung lag bei 31 % (Abb. 1).

### Delphi-Runde 2

**Figure 2:**
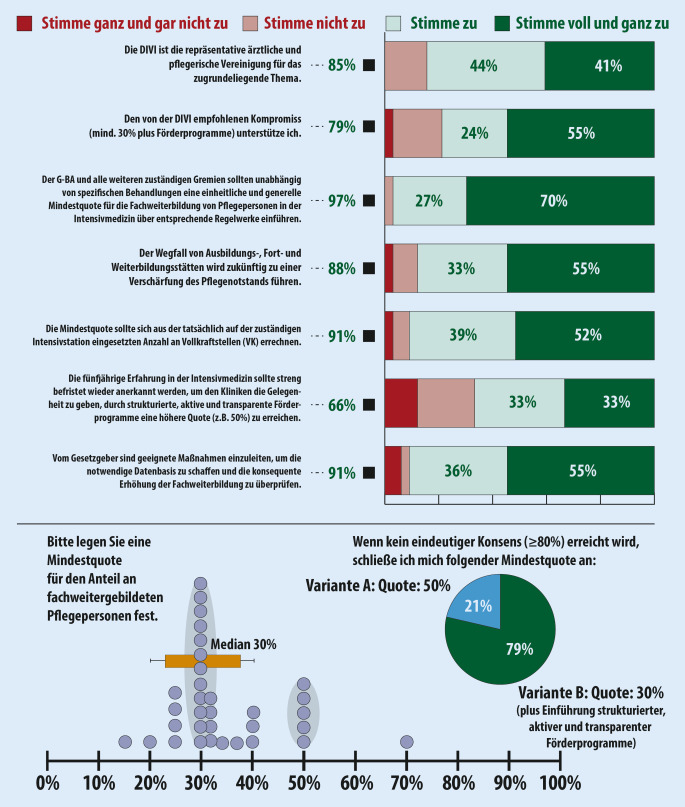

Zur Teilnahme an der zweiten Delphi-Abstimmungsrunde wurden 37 Expert:innen eingeladen (Teilnahmequote 89 %, *n* = 33). Die Erhöhung der Anzahl gegenüber der ersten Runde ergibt sich aus der verspäteten Benennung einer/eines Delegierten aus einem Pflegefachverband. Insgesamt wurden 7 Statements durch das Expert:innenpanel beurteilt, wobei 5 Statements mit über 80 % Zustimmung bewertet wurden (Abb. 2):

- Die DIVI ist die repräsentative ärztliche und pflegerische Vereinigung für das zugrunde liegende Thema. (Zustimmung: 85 %)
- Der G‑BA und alle weiteren zuständigen Gremien sollten unabhängig von spezifischen Behandlungen eine einheitliche und generelle Mindestquote für die Fachweiterbildung von Pflegefachpersonen in der Intensivmedizin über entsprechende Regelwerke einführen. (Zustimmung: 97 %)
- Der Wegfall von Ausbildungs‑, Fort- und Weiterbildungsstätten wird zukünftig zu einer Verschärfung des Pflegenotstands führen. (Zustimmung: 88 %)
- Die Mindestquote sollte sich aus der tatsächlich auf der zuständigen Intensivstation eingesetzten Anzahl an Vollkraftstellen (VK) errechnen. (Zustimmung: 91 %)
- Vom Gesetzgeber sind geeignete Maßnahmen einzuleiten, um die notwendige Datenbasis zu schaffen und die konsequente Erhöhung der Fachweiterbildung zu überprüfen. (Zustimmung: 91 %)

Auf die Frage, welche Mindestquote für den Anteil an fachweitergebildeten Pflegefachpersonen festgelegt werden solle, ergab sich ein Median von 30 %. Mit einer Zustimmung in Höhe von 79 % der Expert:innen verfehlte der von der DIVI vorgeschlagene Kompromissvorschlag die erforderliche Konsensgrenze – wobei die restlichen 21 % der Teilnehmer in diesem Fall („kein eindeutiger Konsens“) für eine höhere Fachweiterbildungsquote von 50 % votierten (Abb. 2).

### Delphi-Runde 3

**Figure 3:**
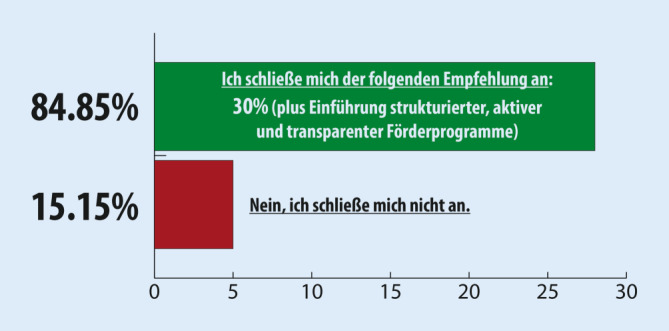

Zur Teilnahme an der dritten Delphi-Abstimmungsrunde wurden 37 Expert:innen eingeladen (Teilnahmequote 89 %, *n* = 33). Der von der DIVI vorgeschlagene Kompromiss erreichte in dieser Runde eine Zustimmung in Höhe von 84,9 % (Abb. 3).

## Diskussion

In diesem modifizierten Delphi-Verfahren mit 37 Expert:innen wurde die Fragestellung diskutiert, welche Mindestquote für die Fachweiterbildung von Pflegefachpersonen der Intensivstation bei der Behandlung des BAA gefordert werden sollte.

Derzeit existieren nach GKV-Recht für Pflegefachpersonen der Intensivstation verbindliche Mindestquoten lediglich für die Leistungsbereiche BAA (50 %), minimal-invasive Herzklappenintervention (25 %) und Neonatologie (40 %). Die Festlegung der Quote für den in diesem Delphi-Verfahren relevanten Leistungsbereich erfolgte bei dem Inkrafttreten der Richtlinie im Jahr 2008 auf dem Boden indirekter Evidenz und Expert:innenmeinungen. Damit ist gemeint, dass keine vergleichende Datenbasis aus Beobachtungs- oder interventionellen Studien verfügbar war, die für die Untersuchung eines direkten Zusammenhangs zwischen der Fachweiterbildungsquote und passenden Endpunkten geeignet war. Die beteiligten Expert:innen haben vor dem Hintergrund der mittlerweile geänderten Versorgungsrealität eine Reevaluation und Anpassung als notwendig erachtet.

In der zusammenfassenden Beurteilung aller Erwägungsgründe und Argumente der beteiligten Expert:innen hat das Panel eine schichtbezogene Mindestquote in Höhe von 30 % der Pflegefachpersonen sowie die Einführung geeigneter Förderprogramme zur langfristigen Erhöhung der Quote auf mindestens 50 % empfohlen. Dabei wurde mit großer Zustimmung auch betont, dass derartige Struktur- und Prozessqualitätsparameter grundsätzlich nicht individuell für einzelne Leistungsbereiche, sondern generell für alle Intensivbehandlungen gelten sollten. Die Mindestquote sollte sich dabei aus der tatsächlich auf der zuständigen Intensivstation eingesetzten Anzahl an Vollkraftstellen errechnen. Als zentraler Ausgangspunkt vieler Diskussionen galten dabei die ehemals auf 50 % festgelegte Quote und der Umstand, dass diese Quote durch die DIVI auch 14 Jahre nach der Einführung der Richtlinie als „aktuell nicht zeitnah umsetzbar“ bewertet wurde [48]. Interessant ist diese Feststellung vor dem Hintergrund, dass andere Länder bereits derartige Quoten eingeführt haben. Allerdings gelten die Vorgaben dort unbeschränkt für alle Intensivbehandlungen und die einwohnerbezogene Anzahl an Intensivbetten ist nach Angaben der OECD um den Faktor 3 (Schweiz, Neuseeland) bis 10 (England) niedriger als in Deutschland. Gleichermaßen haben insbesondere die Expert:innen aus den Bereichen Gefäßchirurgie und Radiologie betont, dass die überwiegende Mehrheit der elektiven Fälle heutzutage mittels EVAR behandelt wird. In aktuellen Analysen von Krankenkassen- und Registerdaten beträgt der Anteil heutzutage demnach bis zu 80 %, während in der Zeit der Einführung der Richtlinie noch etwa 41 % der elektiven Fälle und 77 % der Rupturen offen-chirurgisch behandelt wurden [9, 14]. Interessanterweise wird dieses Verfahren in den gängigen Praxisleitlinien nicht mehr als Hochrisikoeingriff bewertet und führt nur noch in 60 % zu einer postinterventionellen Intensivbehandlung [27, 41]. Es muss allerdings festgehalten werden, dass die Expert:innen auch auf dem Boden indirekter Evidenz, z. B. zum Pflegeschlüssel oder Qualifikationsmaßnahmen, eine höhere Quote grundsätzlich begrüßen. Der hier festgelegte Konsens entspricht somit dem Kompromiss aus erwartbarer Effektivität und Umsetzbarkeit.

Kritisch gesehen wurde die Tatsache, dass die Krankenhäuser und das Gesundheitssystem in Deutschland bereits seit der Einführung der Richtlinie im Jahr 2008 nicht in der Lage waren, die Voraussetzungen für deren flächendeckende Erfüllung sicherzustellen. Durch den sich zuspitzenden Fachkräftemangel, ungünstige Motivatoren und den potenziellen Wegfall weiterer Aus- und Weiterbildungsstätten erscheine es unwahrscheinlich, dass zeitnah flächendeckend höhere Quoten erreichbar sind. Vom Gesetzgeber sind nach Ansicht des Expert:innenpanels daher geeignete Maßnahmen einzuleiten, um die notwendige Datenbasis zu schaffen und die Erhöhung der Fachweiterbildung zu fördern.

Ein erster Schritt wäre die Analyse der Qualitäts- und Versorgungsdaten, die z. B. durch das INEK oder DIMDI vorgehalten werden. Aber auch die wissenschaftliche Auswertung und ggf. Erweiterung der Datenerhebungen des DIGG sowie der Medizinischen Dienste könnten helfen, den Erfolg der eingeleiteten Maßnahmen mittelfristig zu evaluieren.

Vor dem Hintergrund der unzureichenden direkten Evidenzbasis zur Fragestellung ist die Generierung von Empfehlungen durch Expert:innenmeinungen die beste verfügbare Alternative. Hierbei ist allerdings zu betonen, dass Expert:innenmeinungen auch in strukturiert erhobener Form grundsätzlich von der Zusammensetzung des Panels beeinflusst werden [47]. Obwohl Maßnahmen zur Einbeziehung aller relevanten Fachbereiche, unterschiedlicher Krankenhausträger und Schnittstellenbereiche unternommen wurden, ist eine Verzerrung nicht auszuschließen. Durch eine transparente Dokumentation, Kommunikation und anonymisierte Kommentierung des Verfahrens soll sichergestellt werden, dass alle Meinungen und Argumente vom Panel diskutiert werden.

Die sinkende Prävalenz des BAA führt bei den etwa 500 beteiligten Kliniken zwangsläufig zu einer Konkurrenzsituation, der durch Zentralisierungsvorhaben zusätzlich Vorschub geleistet wird. Es erscheint daher naheliegend, dass Einrichtungen verschiedener Versorgungsstufen, insbesondere im Bereich der Intensivmedizin, unterschiedliche Sichtweisen auf diese Fragestellungen haben. Allerdings ist der Pflegekräftemangel vermutlich in strukturschwachen Regionen der Flächenversorgung weniger ein Problem als in Metropolregionen mit bettenreichen intensivmedizinischen Abteilungen. Diese komplexe Interessenlage bei den Versorgungseinrichtungen erfasst dabei nur im Ansatz die zentralen Interessen der betroffenen Patient:innen und Angehörigen, die naheliegenderweise eine möglichst hohe bzw. vollständige Fachweiterbildungsquote einfordern. In jedem Fall erscheint es notwendig, alle Perspektiven einzubeziehen. Auch die Sichtweise der für die Berufsqualifizierung und Fortbildung verantwortlichen Pflege- und Pflegeforschungsverbände gewähren Einblicke, die in diesem Expert:innenverfahren einen wichtigen Stellenwert hatten und die prozedurfokussierte Perspektive ergänzen konnten.

Die rasche Verbreitung endovaskulärer zunehmend weniger invasiver Techniken und die Verfügbarkeit besserer ambulanter Nachsorgestrukturen hatte bereits in den vergangenen Jahren deutliche Auswirkungen auf die Behandlung des BAA. Die alleinige Fokussierung qualitätssichernder Maßnahmen auf die intensivmedizinischen Aspekte dieser prophylaktischen Gefäßintervention erscheint daher anachronistisch und gibt Anlass zu Diskussionen [21]. Auf der anderen Seite ist etwa die technisch herausfordernde und mit höheren Komplikationsraten assoziierte komplexe endovaskuläre Behandlung thorakoabdomineller Aortenerkrankungen oder des Aortenbogens nicht reguliert, was die Notwendigkeit der Überprüfung der in der QBAA-RL festgelegten Fachweiterbildungsquote und insbesondere die Forderung nach leistungsgruppenübergreifenden Quoten zusätzlich unterstreicht.

In einer ersten vorübergehenden Maßnahme und als Reaktion auf die Gefährdung der flächendeckenden Versorgungssicherheit hat der G‑BA am 16.11.2023 einen Beschluss über eine Änderung der QBAA-RL erlassen, nach dem die 5‑jährige Erfahrung in der Intensivmedizin bis Ende 2024 wieder anstelle der Fachweiterbildung treten kann. Es bleibt abzuwarten, inwiefern die Ergebnisse des Delphi-Verfahrens weitere Beschlüsse anstoßen werden.

## Schlussfolgerung

In diesem nationalen Delphi-Verfahren mit ärztlichen und pflegerischen Expert:innen sowie Patientenvertreter:innen wurde der grundsätzliche Nutzen und Bedarf der beruflichen Fachqualifikation im Bereich der Intensivmedizin bestätigt. Die entsprechenden Mindestquoten für eine Fachweiterbildung von Intensivpflegefachpersonen sollten demnach ohne Einschränkung auf spezifische Leistungsgruppen generell gelten. Angesichts der zeitnah nicht realisierbaren Entspannung auf dem Arbeitsmarkt wurde durch das Expert:innenpanel eine schichtbezogene Mindestquote in Höhe von 30 % der Pflegefachpersonen auf der Intensivstation und die verpflichtende Einführung strukturierter und transparenter Förderprogramme zu deren langfristiger Erhöhung auf mindestens 50 % gefordert.

## Fazit für die Praxis

- Während die Behandlung des Bauchaortenaneurysmas kurz nach der Jahrtausendwende noch überwiegend offen-chirurgisch durchgeführt wurde, macht der Anteil an katheterbasierten endovaskulären Verfahren bei der elektiven Behandlung heute bis zu 80 % aus.
- Im Jahr 2008 wurde eine Qualitätssicherungsrichtlinie des Gemeinsamen Bundesausschuss (G-BA) zur Behandlung von Patient:innen mit Bauchaortenaneurysma erlassen, in der auch die Erreichung einer Fachweiterbildungsquote in Höhe von 50 % bei den Pflegefachpersonen der Intensivstation der Einrichtung gefordert wird.
- Aufgrund der aktuellen Versorgungsrealität, der verfügbaren Evidenz und Expertenmeinungen empfiehlt das Expertenpanel die Einführung einer schichtbezogenen Mindestquote in Höhe von 30 % und die gleichzeitige Einführung strukturierter, transparenter und aktiver Förderprogramme zur langfristigen Erhöhung der Quote auf mindestens 50 %.
- Anforderungen an die Struktur- und Prozessqualität von Intensivstationen sollten nicht individuell für einzelne Leistungsgruppen, sondern für die Intensivmedizin insgesamt gelten.
- Es existiert indirekte Evidenz für einen Zusammenhang zwischen dem Skill-Mix des Pflegeteams der Intensivstation und einem besseren Behandlungsergebnis. Eine direkte oder hochwertige Evidenzbasis für einen Zusammenhang zwischen der Fachweiterbildung und dem Behandlungsergebnis existiert bisher nicht, sodass Empfehlungen auf dem Boden von Expertenmeinungen generiert werden müssen.
- Die Krankenhäuser haben es seit 2008 nicht geschafft, entsprechende Programme umzusetzen. Die Erreichung einer Mindestquote in Höhe von 50 % erscheint weiterhin aktuell nicht zeitnah umsetzbar.
- Die Deutsche Interdisziplinäre Vereinigung für Intensiv- und Notfallmedizin (DIVI) ist die ärztliche und pflegerische Vertretung für das Thema der beruflichen Qualifikation in der Intensivmedizin.
- Die Anpassung der Mindestquote sollte durch umfassende Maßnahmen von Politik, Gesundheitswirtschaft und Regulation begleitet werden, um eine Erhöhung der Verfügbarkeit von fachweitergebildeten Pflegefachpersonen zu erreichen.

### Supplementary Information




