# Supplementary material for: Recommendations for the specialist further training of nursing personnel on intensive care units in the treatment of abdominal aortic aneurysms: results of a modified Delphi procedure with experts
Source: Chirurgie (Heidelb). 2024 Mar 18;95(5):395–405. [Article in German] doi: 10.1007/s00104-024-02066-1 (PMC11031449; doi:10.1007/s00104-024-02066-1)
Supplement: Supplementary file 1 [file 104_2024_2066_MOESM1_ESM.docx]

Exposé zum modifizierten Delphi-Verfahren
mit Expert:innen

des Deutschen Instituts für Gefäßmedizinische Gesundheitsforschung gGmbH (DIGG)


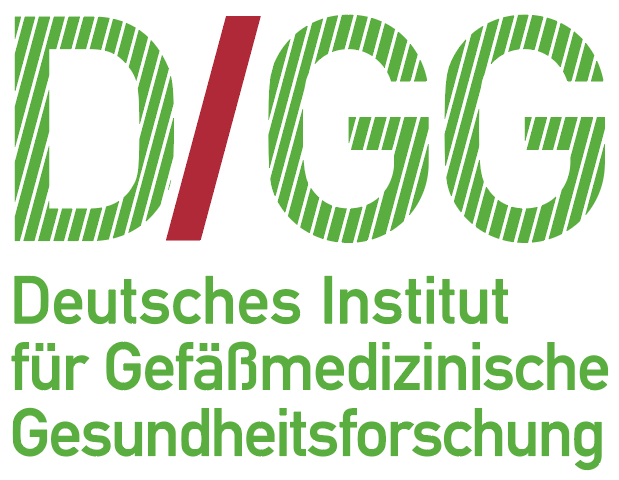


Stand: 20. November 2023

**Zum verantwortlichen Forschungsinstitut:**

Das Deutsche Institut für Gefäßmedizinische Gesundheitsforschung gGmbH (DIGG) ist das wissenschaftliche Institut der Deutschen Gesellschaft für Gefäßchirurgie und Gefäßmedizin - Gesellschaft für operative, endovaskuläre und präventive Gefäßmedizin e.V. (DGG). Das DIGG wurde 2023 durch die Europäische Kommission als Forschungsinstitut akkreditiert und führt nationale und internationale Projekte der Versorgungsforschung und Qualitätsentwicklung in der interdisziplinären Gefäßmedizin durch. Das DIGG ist unter anderem verantwortlich für die Qualitätssicherung zur Behandlung des Bauchaortenaneurysmas an den von der DGG zertifizierten Gefäßzentren und veröffentlicht jährliche Benchmarking- und Behandlungsberichte zu bis zu 200 deutschen Krankenhäusern. Es hat seinen ständigen Geschäftssitz in Berlin und wird gemeinsam durch einen medizinisch-wissenschaftlichen Direktor (Priv.-Doz. Dr. med. Christian-Alexander Behrendt) und eine Geschäftsführerin (Dr. med. Livia Cotta) geleitet (https://www.digg-dgg.de/).^[[1]](#footnote-1)^

**Hintergrundinformationen zum Konsensusverfahren und zur Methodik:**

Das hier beschriebene Konsensusverfahren wurde im Sommer 2023 initiiert, um dem Gemeinsamen Bundesausschuss (G-BA) bei der Diskussion und Abstimmung der sogenannten Intensivpflegequote in der Richtlinie über Maßnahmen zur Qualitätssicherung für die stationäre Versorgung bei der Indikation Bauchaortenaneurysma (QBAA-RL) zu unterstützen. In dieser Richtlinie wird unter anderem gefordert, dass mindestens 50% der Mitarbeiterinnen und Mitarbeiter des Pflegedienstes eine Fachweiterbildung im Bereich Intensivpflege und Anästhesie gemäß der Empfehlung der Deutschen Krankenhausgesellschaft (DKG) aufweisen müssen.^[[2]](#footnote-2)^ Für die Festlegung dieser Quote existierte bei der Erstellung der Richtlinie im Jahr 2008 keine empirische Datenbasis, weshalb sie als Expertenmeinung zu bewerten ist. Die substanziell geänderte Versorgungsrealität im Jahr 2023 und der Fachpflegemangel machen aus Sicht der Initiatoren dieses Konsensusverfahrens eine Reevaluation dieser Quote erforderlich.

Zur Findung eines Konsensus wurden Expert:innen zur Teilnahme eines Delphi-Verfahrens eingeladen. Die Delphi-Methode wurde in den 1950er Jahren durch die RAND-Corporation entwickelt, um komplexe Fragestellungen auf pragmatischem Weg durch ein Expertenpanel zu lösen. Obwohl es bis heute keine einheitliche Vorgabe zur Durchführung von Delphi-Verfahren gibt, existieren typische Charakteristika: Eine für die Fragestellung geeignete Gruppe von Expert:innen beantwortet anonyme Fragebögen in mehreren aufeinander folgenden Runden, zwischen denen jeweils eine moderierte Diskussion und schrittweise Annäherung an den Gruppenkonsens erreicht wird.^[[3]](#footnote-3)^ Die Zusammenstellung des Panels ist dabei maßgeblich für die Beleuchtung der verschiedenen Erwägungsgründe und Argumente.

**Die Grunderkrankung, Zielpopulation und Behandlungsverfahren:**

Die Richtlinie über Maßnahmen zur Qualitätssicherung für die stationäre Versorgung bei der Indikation Bauchaortenaneurysma (QBAA-RL) betrifft die Versorgung von Patientinnen und Patienten mit offen-chirurgisch oder endovaskulär behandlungsbedürftigem Bauchaortenaneurysma. Anlage 1 zur Richtlinie beinhaltet in ihrer aktuellsten Fassung die ICD-10-Codes in der Version 2023: I71.02 (**Dissektion** der Aorta abdominalis, ohne Angabe einer Ruptur) sowie I71.4 (**Aneurysma** der Aorta abdominalis, ohne Angabe einer Ruptur) sowie die OPS-Prozedurencodes 5-384.5*, 5-384.6* und 5-384.7* (Resektion und Ersatz/Interposition an der Aorta) und 5-38a.c*, 8-84a.*4 und 8-84b.*4 (Endovaskuläre Implantation von Stentprothesen, EVAR).


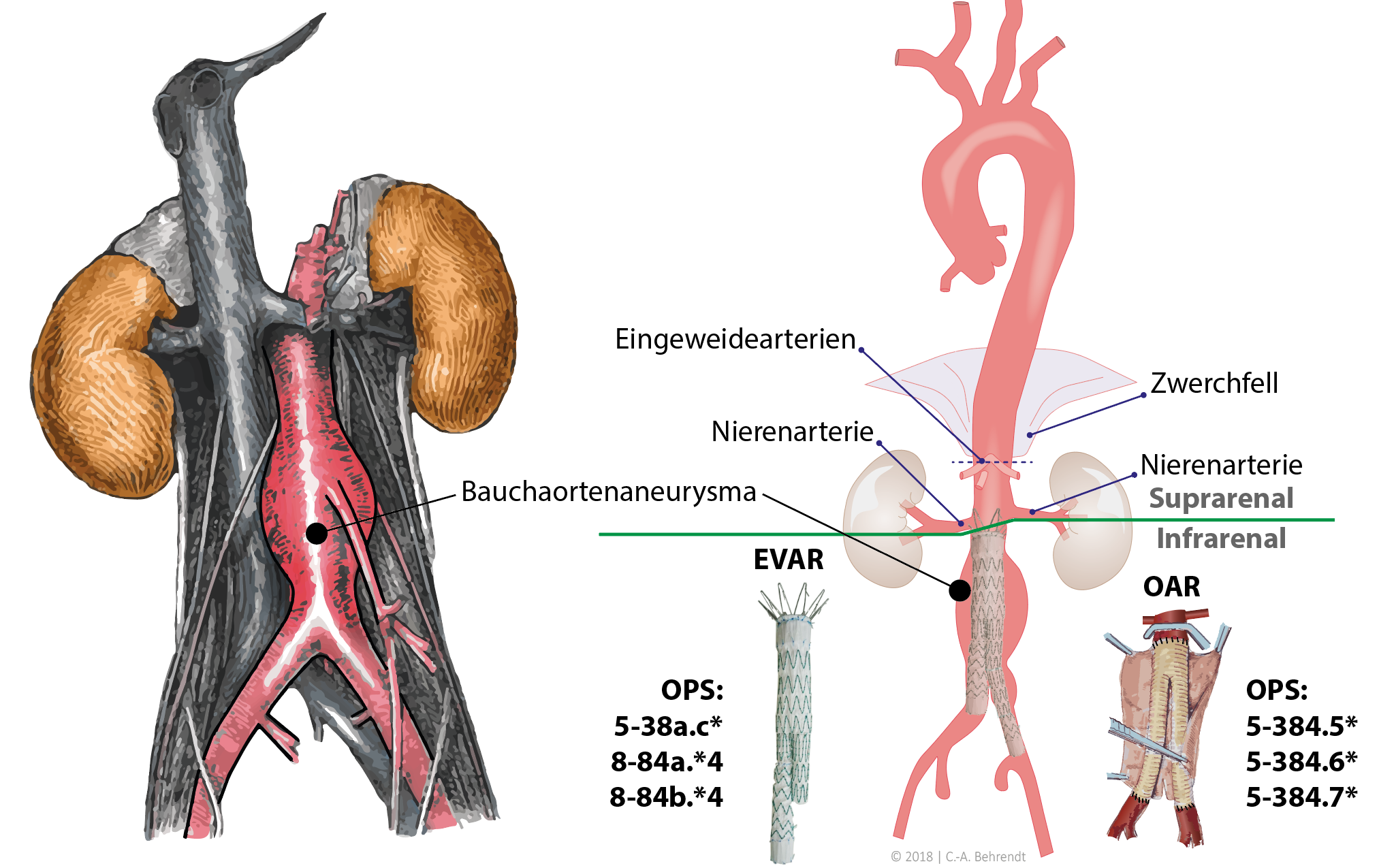


Im klinischen Alltag wird ab 3.0 cm maximalem Durchmesser der abdominalen Aorta von einem Bauchaortenaneurysma gesprochen. Die Häufigkeit dieser krankhaften Erweiterung der Hauptschlagader liegt nach aktuellen epidemiologischen Kohortenstudien bei ca. 0.2% der in Deutschland lebenden Frauen und 1.3% der Männer im medianen Alter von 63 Jahren mit einer statistisch signifikanten Assoziation zu höherem Alter, Rauchen und vorhergegangenen Herzinfarkten.^[[4]](#footnote-4)^ Eine elektive invasive Behandlungsindikation liegt nach internationalen Leitlinienempfehlungen ab einem Durchmesser von 5.5 cm (Männer) bzw. 5.0 cm (Frauen) vor, wobei das primäre Therapieziel die Vermeidung einer meist tödlichen Ruptur ist (prophylaktischer Eingriff).^[[5]](#footnote-5)^ In Deutschland werden derzeit etwa 10.700 nicht rupturierte (intakte) und 1.200 rupturierte Bauchaortenaneurysmen pro Jahr an etwa 500 Krankenhäusern operiert bzw. katheterbasiert interveniert. Dabei zeigt sich seit Jahren eine deutliche Zunahme der endovaskulären Behandlung mittels Stentprothesen (EVAR, *Endovascular Aortic Repair*), deren Anteil derzeit fast 80% der nicht rupturierten Aneurysmen ausmacht.^[[6]](#footnote-6)^ Dieser Entwicklung liegen vielfältige Faktoren zugrunde, wobei die randomisierten kontrollierten Studien zwar einen kurz- und mittelfristigen Vorteil für EVAR belegten, der im längerfristigen Verlauf nach 2-3 Jahren allerdings aufgehoben wurde.^[[7]](#footnote-7)^ Laut den Routinedaten der Krankenkassen, Qualitätsregisterdaten des DIGG und Krankenhausdiagnosestatistiken des Bundes starben während des etwa ein bis zwei Wochen langen Krankenhausaufenthaltes etwa 1-2% nach EVAR bzw. 5-6% nach offen-chirurgischer Versorgung. Zu den häufigeren Komplikationen zählten dabei die akute Extremitätenischämie, der akute Myokardinfarkt und die akute Mesenterialischämie.^[[8]](#footnote-8),^^[[9]](#footnote-9),^^[[10]](#footnote-10)^ In einer weiteren internationalen Routinedatenanalyse aus Deutschland, Australien und den USA betrug die kurzfristige Sterblichkeit innerhalb von 60 Tagen nach EVAR zwischen 1.9% in Australien und 2.9% in Deutschland und nach offen-chirurgischem Aortenersatz zwischen 5.5% in Australien und 8.4% in Deutschland.^7^

In Deutschland werden derzeit etwa 60% der Patient:innen nach EVAR und 96% nach offen-chirurgischem Aortenersatz postoperativ vorübergehend auf einer entsprechenden Monitor- oder Intensivstation überwacht.^10^ In den Qualitätssicherungsdaten des DIGG gehörten dabei die Verschlechterung der Nierenfunktion (1.3%), Blutungen der Zugangsgefäße (1.0%) und Lungenentzündungen (1.0%) zu den häufigsten akuten Komplikationen, während Harnwegsinfekte (0.8%), respiratorische Insuffizienz (0.7%) und schwere kardiopulmonale Komplikationen insgesamt sehr selten waren. Nach offen-chirurgischer Versorgung kam es deutlich häufiger zu einer Pneumonie (9.2%) oder respiratorischen Insuffizienz (6.5%), zu einer Verschlechterung der Nierenfunktion (7.8%) mit Dialysepflichtigkeit (4.3%) oder Darmischämie (3.7%).^10^

**Indikatoren der Behandlungsqualität in der Qualitätssicherungsrichtlinie BAA:**

Die Richtlinie über Maßnahmen zur Qualitätssicherung für die stationäre Versorgung bei der Indikation Bauchaortenaneurysma (QBAA-RL) beinhaltet verschiedene Empfehlungen und Erwägungen zu Struktur-, Prozess- und Ergebnisindikatoren. Neben den allgemeinen Strukturanforderungen an die nach §108 SGB V zugelassenen Krankenhäuser gelten spezifische Anforderungen. So muss die fachliche Leitung und eine weitere Ärzt:in über die Facharztanerkennung Gefäßchirurgie verfügen und für die endovaskuläre Therapie muss eine entsprechende Expertise (alleine oder in Kooperation) nachgewiesen werden. Sowohl die stationäre postprozedurale Versorgung als auch der Bereitschaftsdienst müsse (innerhalb von 30 Minuten) durch Gefäßchirurg:innen wahrgenommen werden. Für die Narkosedurchführung wird eine fachanästhesiologische Betreuung durch erfahrene Ärzt:innen gefordert. Gleichermaßen wird für die präoperative Diagnostik ein interdisziplinäres Facharztteam unter Einbeziehung der Kerndisziplinen Gefäßchirurgie, Radiologie, Innere Medizin (Kardiologie), Anästhesiologie und Labormedizin gefordert. Als operative und intensivmedizinische Ausstattung wird ein moderner Operationssaal, eine Intensivstation in räumlicher Nähe zum Operationssaal mit der Möglichkeit der Behandlung von Multiorganversagen und eine labormedizinische bzw. transfusionsmedizinische Vorhaltung gefordert.

**Vorgaben zur Pflegequote in der Qualitätssicherungsrichtlinie BAA:**

Zum Pflegedienst der Intensivstation der Einrichtung legt §4 Abs. 3 der Richtlinie fest:

„Der Pflegedienst der Intensivstation der Einrichtung gemäß § 1 Abs. 2 muss aus Gesundheits- und Krankenpflegerinnen oder Gesundheits- und Krankenpflegern bestehen. **50%** der Mitarbeiterinnen und Mitarbeiter des Pflegedienstes müssen eine Fachweiterbildung im Bereich Intensivpflege und Anästhesie gemäß der Empfehlung der Deutschen Krankenhausgesellschaft („DKG-Empfehlung zur Weiterbildung für Krankenpflegepersonen in der Intensivpflege“ vom 11. Mai 1998 oder „DKG-Empfehlung zur Weiterbildung von Gesundheits- und (Kinder-)Krankenpflegekräften für die pflegerischen Fachgebiete Intensivpflege, Funktionsdienste, Pflege in der Onkologie, Nephrologie und Psychiatrie“ vom 20. September 2011) oder einer gleichwertigen landesrechtlichen Regelung abgeschlossen haben. Die DKG gibt zur Gleichwertigkeit der einzelnen landesrechtlichen Regelungen jeweils eine Einschätzung ab. Es muss **in jeder Schicht eine Pflegekraft** mit Fachweiterbildung im Bereich Intensivpflege und Anästhesie eingesetzt werden. Anstelle der Fachweiterbildung in den Sätzen 2 und 3 kann bis zum 31. Dezember 2015 jeweils eine mindestens fünfjährige Erfahrung in der Intensivpflege treten.

Die Stationsleitung hat zusätzlich einen **Leitungslehrgang** absolviert“

**Die verfügbare Evidenzbasis und Erfahrungen in anderen Ländern:**

In einer informellen Umfrage unter Repräsentant:innen der Teilnehmerländer im VASCUNET Qualitätsentwicklungs- und Registerkonsortium der European Society for Vascular Surgery (ESVS, www.vascunet.org) und des International Consortium of Vascular Registries (ICVR)^[[11]](#footnote-11),^^[[12]](#footnote-12)^ wurde rückgemeldet, dass zum Zeitpunkt der Befragung **in keinem der anderen Teilnehmerländer** (u.a. Schweden, Dänemark, Spanien, USA, Malta, Ungarn, Österreich, Finnland) für die Behandlung von Bauchaortenaneurysmen eine verbindliche Mindestquote festgelegt wurde. Teilweise existieren allerdings länderspezifisch erweiterte Aus- und Weiterbildungscurricula sowie kompetenzbasierte Qualifikationsmöglichkeiten für Pflegepersonen auf Intensivstationen, die nicht dezidiert für die Behandlung von Patient:innen mit Bauchaortenaneurysma gelten. In der Schweiz (umfasst etwa 70 Spitäler mit Intensivstationen) gilt beispielsweise eine generelle vom Bauchaortenaneurysma unabhängige Zertifizierungsvorgabe für Intensivstationen. Demnach soll mindestens ein Drittel der dort tätigen Pflegepersonen ein anerkanntes Diplom „Experte in Intensivpflege NDS HF“ oder eine gleichwertige Ausbildung verfügen. Sowohl das Diplom als auch die Anerkennung der Gleichwertigkeit erfolgen dort durch die zuständige Fachgesellschaft.^[[13]](#footnote-13)^ In den USA (umfasst mehr als 2.000 Einrichtungen mit Intensivstationen) gilt eine generelle Mindestvoraussetzung für die Pflegetätigkeit auf Intensivstationen von vier Jahren (Bachelor of Nursing Degree) sowie zwei Jahren praktischer Ausbildung mit Abschluss gefolgt von „Erfahrung auf Intensivstationen“ (variabel). In England (umfasst ca. 300 Einrichtungen und Netzwerke) existieren unspezifische Standards für die Intensivpflegepersonen im Bereich von „Critical Care Services“ (z.B. pädiatrische Intensivmedizin, Verbrennungsmedizin, ECMO-Service), wobei mindestens 50% der Pflegepersonen eine ausbildungsergänzende akademische Fortbildung in „Critical Care Nursing“ nachweisen müssen.^[[14]](#footnote-14)^

Hochwertige vergleichende randomisierte Evidenz (Level IA-Evidenz) zur Wirksamkeit, Sicherheit und Kosteneffizienz von Weiterbildungsquoten bei Pflegepersonen bei der Behandlung des Bauchaortenaneurysmas liegt bisher nicht vor und auch die verfügbaren Beobachtungsstudien zu weiteren Strukturqualitätsparametern (z.B. Fallvolumen oder Facharztqualifikation) lieferten unterschiedliche Ergebnisse, was sich auch in heterogenen Leitlinienempfehlungen niederschlug. Indirekte Hinweise, deren Übertragbarkeit allerdings zu prüfen wäre, ergaben sich gegebenenfalls aus anderen Fachbereichen, wobei die vom G-BA geforderten Quoten dort teilweise deutlich niedriger ausfallen.

In den aktuellen Empfehlungen zur Struktur und Ausstattung von Intensivstationen 2022 der Deutschen Interdisziplinären Vereinigung für Intensiv- und Notfallmedizin (DIVI), deren Zusammenstellung durch eine DIVI-Expertengruppe konsentiert wurde, finden sich drei Stufen der Basis-, erweiterten und umfassenden Notfallversorgung mit Implikationen für die „pflegerische Qualifikation“ der Intensivstation. Demnach wird bereits in Stufe 1 (flächendeckende Basis-Versorgung) eine Fachweiterbildungsquote >30% des Pflegeteams der Intensivstation und in jeder Schicht gefordert, während in Stufe 2 (erweiterte Versorgung) und Stufe 3 (umfassende Versorgung) **zusätzlich** ein „strukturiertes, aktives und transparentes Förderprogramm“ zur Erhöhung des Anteils auf mindestens 50% empfohlen wird.

Im entsprechenden Hintergrundtext finden sich ausschließlich Empfehlungen auf dem Boden eines Expertenkonsens (Level of Evidence C): *Der „Anteil an Pflegefachpersonen mit der zusätzlichen Fachweiterbildung ‚Intensivpflege und Anästhesie‘ oder ‚Intensivpflege‘ soll in jeder Schicht* ***mindestens 30% des Pflegeteams*** *der Intensivstation betragen (Empfehlungsgrad 1C).* ***Es sollen Maßnahmen ergriffen werden****, den Anteil auf mindestens 50% zu erhöhen (Empfehlungsgrad 1C).“*^[[15]](#footnote-15)^

In der zusammenfassenden Beurteilung der verfügbaren Daten und Expertenmeinungen erscheint es derzeit nicht möglich, eine direkte und evidenzbasierte Aussage zu treffen. Die hilfsweise Nutzung indirekter Evidenz, z.B. aus anderen Behandlungsbereichen oder generellen Empfehlungen zur Struktur- und Prozessqualität der intensivstationären Versorgung, müsste darüber hinaus auf ihre Übertragbarkeit geprüft werden. Der Forderung der DIVI nach einer flächendeckenden Erhöhung der fachweitergebildeten Pflegepersonen auf Intensivstationen auf mindestens 30% steht dabei die derzeitige Realität gegenüber, dass bei anderen Indikationen, z.B. bei minimalinvasiven Herzklappeninterventionen, durch entsprechende G-BA-Richtlinien niedrigere (z.B. 25%) oder bisher keine Mindestquoten gefordert sind (z.B. für komplexe thorako-abdominelle Aorten-Interventionen).

**Bei der Vorbereitung des Delphi-Verfahrens aus dem Panel gesammelte Argumente für oder gegen die Festlegung des Anteils an Fachpflegepersonen mit der zusätzlichen Fachweiterbildung „Intensivpflege und Anästhesie“ bei der Behandlung des Bauchaortenaneurysmas (unvollständig):**

- *Es gibt* ***bisher keine spezifische empirische Datenbasis*** *oder hochwertige Evidenzbasis, die eine Fachpflegequote erklären würde. Selbst die von der DIVI empfohlenen 30% für alle Level der Intensivmedizin sind auf dem Boden einer Expertenmeinung festgelegt worden, wobei auch eingeworfen wurde, dass diese höhere (50%) Quote nicht realistisch und zeitnah erreichbar sei.*
- *Eine Änderung der G-BA-Empfehlung (derzeit 50%) ist* ***nur auf dem Boden einer entsprechenden Evidenzbasis möglich****, wobei auch ein Expertenkonsens als Evidenz gilt. Daher ist ein aktueller Expertenkonsens mit Berücksichtigung der heutigen Versorgungssituation geeignet, diese Änderung zu begründen.*
- *Kleine Kliniken können wegen geringerer* ***Personalfluktuationen*** *die 50%-Quote möglicherweise besser erfüllen als große (Maximal-)Versorger mit sehr komplexer Intensivmedizin.*
- *Der derzeitige* ***Wegfall von Kliniken bei der Versorgung*** *des Bauchaortenaneurysmas könnte in der Fläche zu einer Unterversorgung von Patient:innen und damit zu einem* ***Kollateralschaden*** *führen (es gibt Belege für und gegen eine Mobilität von Patient:innen).*
- *Die jetzt flächendeckende Überprüfung durch den Medizinischen Dienst (MD) hat ergeben (auf dem Boden einer Befragung von DGG-Zentren), dass* ***etwa 30-40% oder sogar mehr diese Richtlinie nicht erfüllen können oder nur über Hilfskonstrukte*** *(z.B. kleiner Untereinheiten) eine adäquate Quote erreichen können. Die Bildung von Untereinheiten kann dabei aber sogar eine bessere Abdeckung (z.B. 100%) ermöglichen, erfordert aber zusätzlichen Aufwand.*
- *Die Diskussion logistischer und administrativer Herausforderungen bei der Personalbeschaffung oder Qualifikation sollten erst sekundär eine Rolle spielen, da es hier um die* ***Patient:innensicherheit*** *geht.*
- *Die* ***Rolle der Krankenhaus- oder 30-Tage-Sterblichkeit*** *als einziger Indikator der Ergebnisqualität bei der elektiven Versorgung des Bauchaortenaneurysmas ist grundsätzlich fraglich.*
- *Im Rahmen der Vorschläge und Diskussionen zur Krankenhausreform wird offensichtlich, dass das Bauchaortenaneurysma als* ***Anwendungsbeispiel für eine Zentralisierung*** *genutzt wird. Warum sollte es für diese Leistungsgruppe (Bauchaortenaneurysma) höhere Anforderungen geben, als für wesentlich komplexere Prozeduren?*
- *Warum sollte über die* ***pro Schicht geforderte Anwesenheit*** *von mindestens einer fachweitergebildeten Pflegeperson und die verpflichtende Anwesenheit von examinierten Gesundheits- und Krankenpflegern mit dem entsprechenden Personalpflegeschlüssel überhaupt eine Quote erforderlich sein? Die* ***Sicherstellung der Qualität der Intensivpflege*** *kann über die fachlich verantwortliche Pflegeperson mit der entsprechenden Qualifikation sichergestellt werden.*
- *Die G-BA-Richtlinie legt die Quote für die Behandlung des Bauchaortenaneurysmas fest und* ***vereinfacht damit die komplexe Versorgungsrealität****. Patient:innen müssen nach Implantation einer EVAR nicht zwingend intensivstationär behandelt werden, während die wesentlich komplikationsträchtigere Versorgung mit fenestrierten und gebranchten Stentprothesen bei thorako-abdominellen Aortenaneurysmen nicht reguliert wird.*
- *Bei der Festlegung von Mindestmengen oder Quoten gilt auch das* ***Gebot der Umsetzbarkeit und Ökonomie****, da wir sonst auch 100% für alle intensivstationären Behandlungen fordern müssten („viel hilft viel“).*
- *Eine* ***Expertenmeinung aus der Zeit vor 2008*** *kann auf die heutige Versorgungsrealität nicht übertragen werden. Der Anteil minimalinvasiver perkutaner Behandlungen mittels EVAR, die verwendeten Medizinprodukte und die medizinische Behandlung hat sich insgesamt deutlich geändert.*
- *Der Wegfall der ersatzweisen „****mindestens fünfjährigen Erfahrung in der Intensivpflege****“ (bis 2015) ist nicht erklärbar. Warum sollte eine langjährige Erfahrung in der Intensivpflege nicht zu der notwendigen Kompetenz führen?*
- *Die postinterventionelle intensivstationäre Behandlung von Patient:innen mit EVAR umfasst, wenn überhaupt, nur die ersten 24 Stunden. Das* ***Komplikationsprofil*** *unterscheidet sich dabei, bis auf die Aortenruptur, nur unwesentlich von koronar-interventionell behandelten Patient:innen (z.B. nach STEMI). Bei der Behandlung mittels offen-chirurgischem Aortenersatz führen die kardio-pulmonalen Komplikationen, die wiederum mit anderen Intensivpatient:innen vergleichbar sind.* ***Eine höhere Rate als bei anderen Richtlinien (z.B. Herzklappeninterventionen, Neonatologie) erscheint daher nicht nachvollziehbar.***
- *Grundsätzlich sollte eine* ***möglichst breite Fort- und Weiterbildung*** *der beteiligten Berufsgruppen unterstützt und sogar gefordert werden.*
- *Bei der Diskussion wird nicht berücksichtigt, dass beim Auftreten von Komplikationen auch eine* ***Weiterverlegung an entsprechende Einrichtungen*** *möglich ist und gelebt wird.*
- *Bei der Diskussion erlangt die* ***Mindestqualifikation der Pflegepersonen ein Übergewicht gegenüber der Arbeit als Team*** *aus Ärzt:innen, Therapeut:innen und Pfleger:innen. Die Diagnostik und Behandlung der häufigsten Komplikationen, z.B. Pneumonie und Niereninsuffizienz, ist nicht überwiegend durch eine Berufsgruppe zu verantworten.*
- *Die Deutsche Gesellschaft für Gefäßchirurgie und Gefäßmedizin (DGG) und andere an der Behandlung der Zielpopulation beteiligte Fachdisziplinen haben solche* ***Fachpflegequoten für die Zertifizierung ihrer Gefäßzentren nie verbindlich festgelegt****, obwohl das Qualitätssicherungsregister zur Behandlung des Bauchaortenaneurysmas dort seit Jahrzehnten alle erforderlichen Daten zusammenträgt.*
- *Zumeist fordern Berufsverbände oder Fachgesellschaften* ***die Umsetzung bzw. Verbreitung eigener Zertifizierungen oder Weiterbildungen****, die allerdings nicht immer staatlich reguliert werden. Aktuell existiert ein Mangel an Fachkräften, um den Bedarf zu decken, weshalb die Regulation des Versorgungsgeschehens im Vordergrund zu stehen scheint.*
- *Umsetzbarkeit von Personalanforderungen* ***unterscheidet sich zwischen den Ländern*** *in Abhängigkeit der Anzahl an Kliniken und Intensivbetten.*
- *Die Erfahrung ist insgesamt schlecht messbar, so dass eine formale Qualifikation einzuordnen wäre (auch damit die verschiedenen Berufsgruppen wissen, mit wem sie zusammenarbeiten).*
- *Wenn wir einen 100% Facharztstandard haben wollen, dann sollten wir auch einen 100% Fachweiterbildungs-Pflegestandard erwarten dürfen.*
- *In der schwierigen Abwägung zwischen wünschenswertem Zielwert (100%) und realisierbarer Quote (Machbarkeit), ist folgender Kompromiss zu empfehlen: Fachweiterbildungsquote > 30 % des Pflegeteams der Intensivstation und in jeder Schicht. Strukturiertes, aktives und transparentes Förderprogramm, um den Anteil auf mindestens 50 % zu erhöhen.*
- *Alle Empfehlungen sollten grundsätzlich unabhängig von Leistungsgruppen, Prozeduren oder Krankheitsbildern für alle intensivmedizinischen Leistungen der entsprechenden Stufen gelten.*
- *Es ist davon auszugehen, dass sich das Problem innerhalb der nächsten fünf Jahre nicht durch Förderprogramme beseitigen lässt, weshalb umfassende durch Politik und Regulation sowie Kostenträger zu finanzierende Maßnahmen erforderlich sind.*
- *Die negative Entwicklung der letzten 15 Jahre hat (insbesondere in NRW) zuletzt zu einer weiteren Reduktion der Weiterbildungsstätten geführt, weshalb sich die Erreichbarkeit in Zeiten des Pflegenotstands noch weiter entfernt.*
- *Eine (vorübergehende) Anerkennung der fünfjährigen Erfahrung in der Intensivmedizin ist schwer definierbar bzw. objektivierbar und sollte auch nur einen vorübergehenden Charakter behalten, daher ist eine erneute Einführung kritisch, weil das Problem damit nicht behoben wird (Mangel an qualifiziertem Personal).*
- *Die DIVI ist die repräsentative ärztliche und pflegerische Vereinigung für dieses Thema.*

**Das Expertenpanel:**

In dem aktuellen Delphi-Verfahren sollen die zahlreichen Erwägungsgründe sowohl vor dem Hintergrund objektivierbarer Kriterien bzw. empirischen Daten als auch subjektiver Expertenmeinungen diskutiert und abgestimmt werden. Dabei sind insbesondere die Argumente der primär an der Versorgung beteiligten Fachdisziplinen (Gefäßchirurgie, Radiologie, Intensivmedizin, Anästhesie), der Berufsgruppen (Ärzt:innen, Pflegende), der beteiligten Schnittstellenbereiche (Qualitätssicherung, Evaluation, Sozialversicherungs- und Kostenträger) als auch der Patient:innen maßgeblich. Es sollten zudem unterschiedliche Krankenhaustypen und -träger eingebunden werden (universitär, privat, kirchlich etc.). Die Rekrutierung der Expert:innen erfolgte breit über die beteiligten Fachgesellschaften und Berufsverbände. Aus dem Bereich Pflege wurden acht Verbände eingebunden.

**Zusammensetzung, Inklusivität, Repräsentativität und Diversität:**

Unter den insgesamt 37 beteiligten Mitgliedern des Expertenpanels (plus 1 wissenschaftliche Moderation) waren sechs Frauen (16%). Insgesamt nahmen 26 (70%) Ärzt:innen und acht (22%) Pflegende teil. Unter den primär ärztlichen Expert:innen waren universitäre (n=11), konfessionelle, private und kommunale Einrichtungen vertreten. Unter den primär pflegerischen Expert:innen waren acht Berufsverbände und Fachgesellschaften vertreten.

Fünf Expert:innen waren hauptamtlich für die Bereiche Krankenhausökonomie, Qualitätssicherung, Kostenträger und medizinischer Dienst, Fachgesellschaften sowie die wissenschaftliche Evaluation in der Gesundheitsversorgung aktiv.

Die vertretenen ärztlichen Fachdisziplinen schlossen primär die Gefäßchirurgie, Anästhesie, Intensivmedizin und Radiologie ein. Die in Deutschland repräsentative pflegerische und ärztliche Vereinigung für Intensivmedizin und Anästhesie (DIVI) war durch zwei Expert:innen vertreten.

**Ablauf dieses modifizierten Delphi-Verfahrens mit Expert:innen:**

| **KW 22-30 (Juni/Juli 2023)** | **Einladung** zur Teilnahme am Expertenpanel an beteiligte Fachgesellschaften und Verbände sowie universitäre und nicht-universitäre Expert:innen; Konstituierung des Panels. |
| --- | --- |
| **KW 31-32 (August 2023)** | Durchführung von 2-3 **Informationsveranstaltungen** (online) für die Mitglieder des Panels, um Fragen zu klären und den Kenntnisstand über die G-BA-Richtlinie und zugrundeliegende Empfehlungen zu harmonisieren. |
| **KW 33 (August 2023)** | Durchführung einer **vorbereitenden Feedbackrunde/Fokusgruppe** (online) zur Sammlung von Argumenten, Erwägungsgründen und Literaturnachweisen (Grauliteratur). |
| **KW 34  (August 2023)** | **Moderierte Diskussion** der Ergebnisse der vorbereitenden Runde. |
| **KW 35  (August 2023)** | Durchführung der **ersten anonymen Abstimmungsrunde** zur Festlegung einer Fachweiterbildungsquote für Pflegepersonen. |
| **KW 36  (September 2023)** | **Moderierte Diskussion** der Abstimmungsergebnisse. |
| **KW 37  (September 2023)** | Durchführung der **zweiten anonymen Abstimmungsrunde** zur Festlegung einer Fachweiterbildungsquote für Pflegepersonen. |
| **KW 38  (September 2023)** | Diskussion und **Konsentierung des Abstimmungsergebnisses**. |
| **KW40**  **(Oktober 2023)** | Durchführung der **dritten anonymen Abstimmungsrunde**  und Diskussion der Ergebnisse |
| **KW 41**  **(Oktober 2023)** | Abschlussauswertung und Information des Expertenpanels über den erreichten Konsensus; Vorabinformation an den G-BA. |
| **Ende Oktober** | **Bereitstellung des Ergebnisses** an die beteiligten Fachgesellschaften und Verbände sowie den Gemeinsamen Bundesausschuss. |

**Abstimmungsergebnisse der ersten Runde (#1) Delphi-Verfahrens mit Expert:innen:**

**
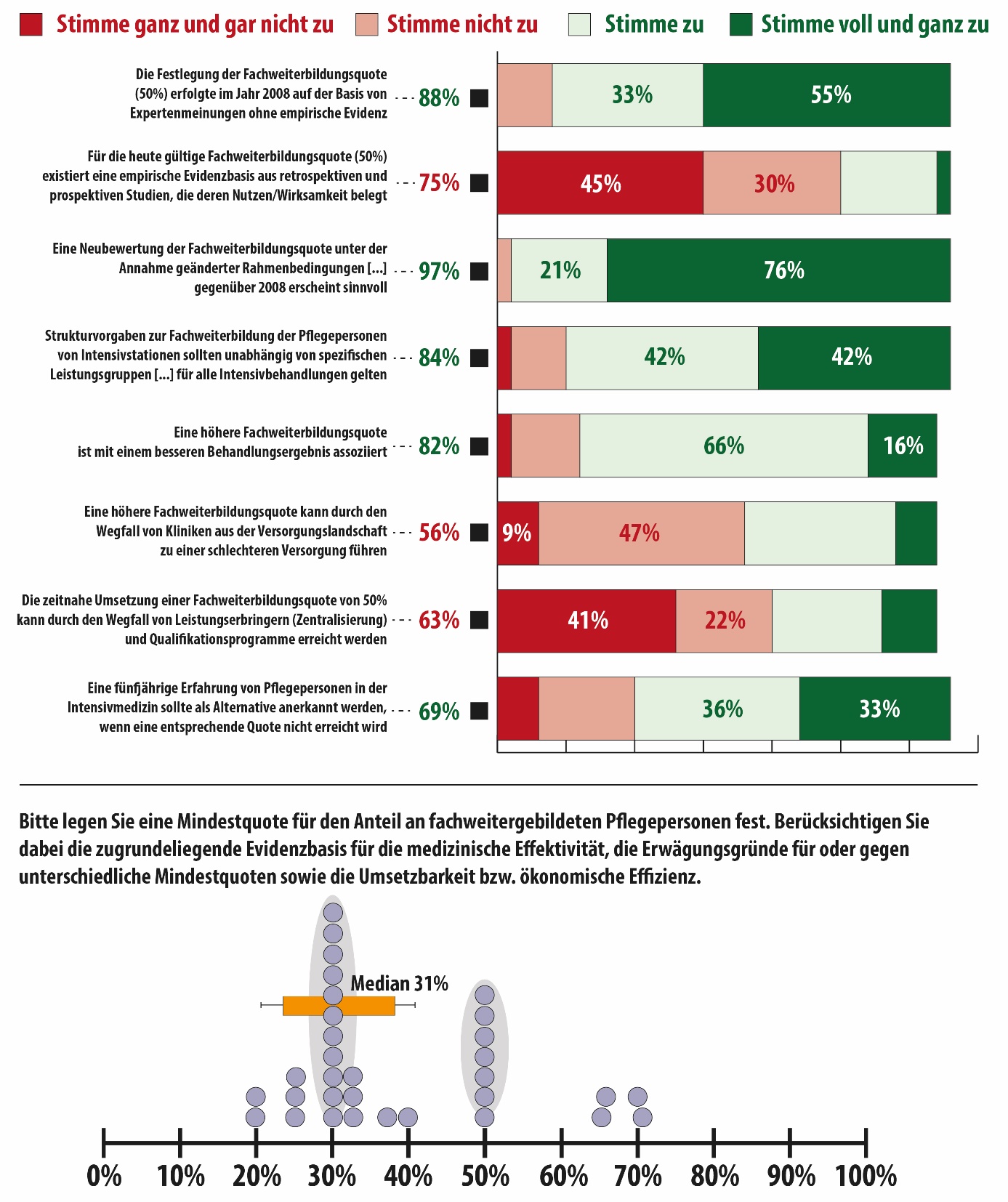
**

**Abstimmungsergebnisse der zweiten Runde (#2) Delphi-Verfahrens mit Expert:innen:**

**
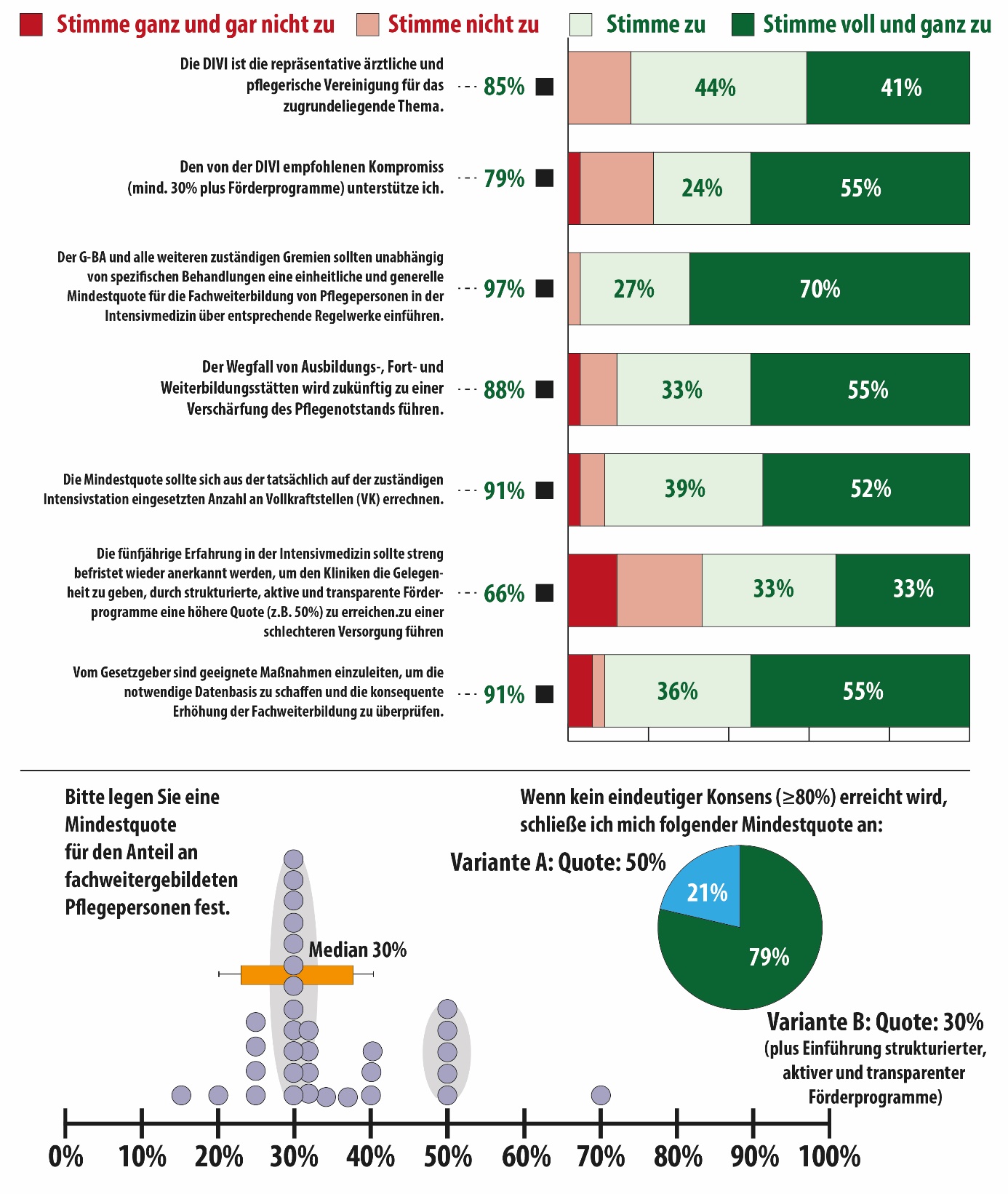
**

**Abstimmungsergebnisse der dritten Runde (#3) Delphi-Verfahrens mit Expert:innen:**

**
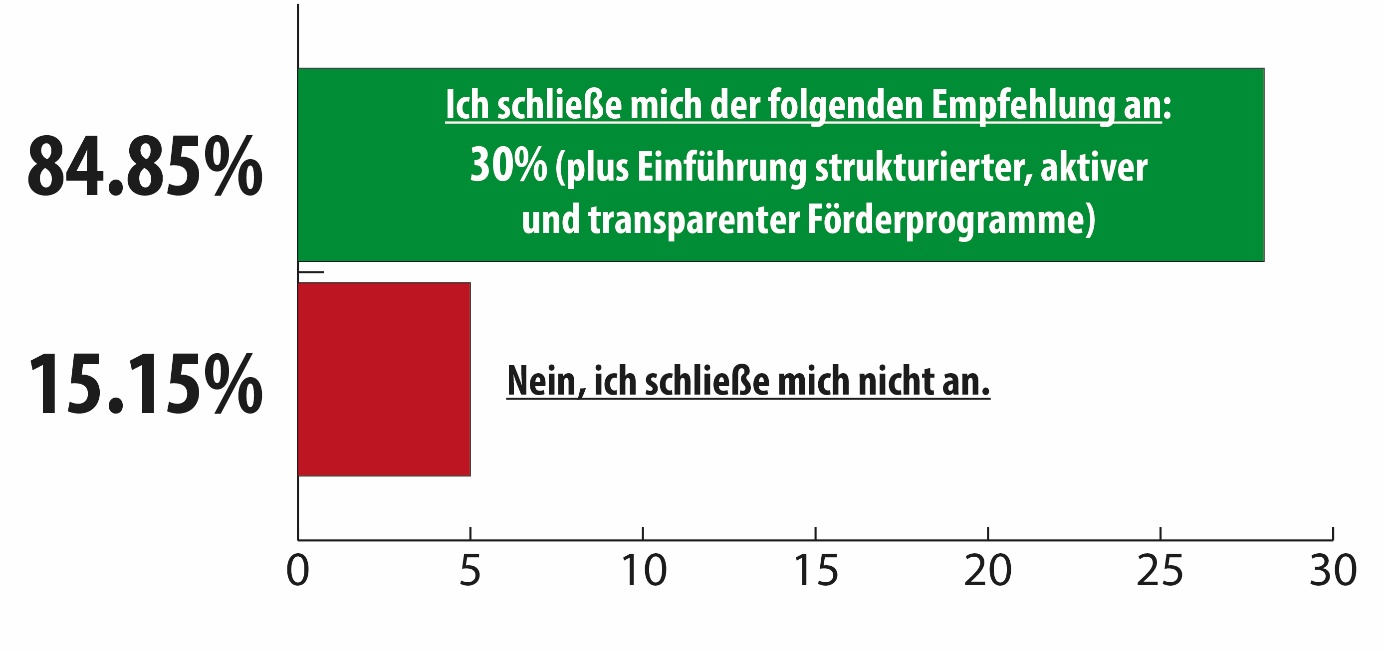
**

**Abschlussbewertung:**

Während der vorbereitenden Diskussionen, den drei Delphi-Abstimmungsrunden und aufeinanderfolgenden Online-Diskussionen wurden die zahlreichen Erwägungsgründe für und gegen bestimmte Mindestquoten für die Fachweiterbildung Intensivmedizin bei Pflegepersonen diskutiert.

Der eher arbiträre Charakter der 2008 gewählten Mindestquote (50%) wurde dabei bestätigt. Obwohl die Mehrheit der Expert:innen vom Nutzen entsprechender Mindestquoten für die Behandlungsqualität überzeugt waren, ist die Umsetzbarkeit der aktuellen Quote gegenwärtig und in mittelbarer Zukunft als unwahrscheinlich bewertet worden. Insbesondere die Versäumnisse der Vergangenheit, die fehlenden Anreize und Risiken (Personalausfall), der Wegfall von Weiterbildungsstätten sowie die derzeitige Entwicklung auf dem Pflegepersonalmarkt (Pflegenotstand) machen gemeinsame Bemühungen und wichtige Schritte durch Gesetzgebung und Gesundheitssystem erforderlich, um langfristig eine höhere Quote zu erreichen.

Der durch die DIVI bereits 2022 erarbeitete Kompromissvorschlag in Höhe von „30% Fachweiterbildung der Pflegepersonen auf der Intensivstation plus eine verbindliche Einführung strukturierter, aktiver und transparenter Förderprogramme“ wurde durch das Expertenpanel konsentiert.

Dieser Kompromissvorschlag soll allerdings nicht davon ablenken, dass unbedingt Maßnahmen erforderlich sind, um die Berufsqualifikation konsequent und nachhaltig zu verbessern.

**Teilnehmende Expert:innen in alphabetischer Reihenfolge**

Christian-Alexander Behrendt^1,2,a^, Jörg Heckenkamp^3^, Andrea Bergsträßer^4^, Arend Billing^5^, Dittmar Böckler^6^, Arno Bücker^7^, Livia Cotta^1^, Konstantinos P. Donas^8^, Gerd Grözinger^9^, Claus-Dieter Heidecke^10^, Irene Hinterseher^11^, Silvio Horn^12^, Arno Kaltwasser^13^, Andrea Kiefer^14^, Claudia Kirnich-Müller^15^, Lars Kock^16^, Tilo Kölbel^17^, Martin Czerny^18,19^, Christian Kralewski^20^, Stephan Kurz^21,22^, Axel Larena-Avellaneda^23^, Haitham Mutlak^24^, Alexander Oberhuber^25^, Kyriakos Oikonomou^26^, Manfred Pfeiffer^27^, Karin Pfister^28^, Christian Reeps^29^, Andreas Schäfer^30^, Thomas Schmitz-Rixen^31^, Markus Steinbauer^32^, Claudia Steinbauer^33^, Daniel Strupp^34^, Dietmar Stolecki^35^, Matthias Trenner^36^, Christof Veit^37^, Eric Verhoeven^38^, Christian Waydhas^39,40^, Christian F. Weber^41,42^, Farzin Adili^43^

^1^ Deutsches Institut für Gefäßmedizinische Gesundheitsforschung, Berlin, Deutschland
^2^ Abt. für Allgemeine und Endovaskuläre Gefäßchirurgie, Asklepios Klinik Wandsbek, Asklepios Medical School, Hamburg, Deutschland
^3^ Niels-Stensen-Kliniken, Osnabrück, Deutschland
^4^ Deutscher Pflegerat e.V., Berlin, Deutschland
^5^ Kommission Krankenhausökonomie, Deutsche Gesellschaft für Gefäßchirurgie und Gefäßmedizin e.V., Berlin, Deutschland
^6^ Klinik für Gefäßchirurgie und Endovaskuläre Chirurgie, Universitätsklinikum Heidelberg, Heidelberg, Deutschland
^7^ Klinik für Diagnostische und Interventionelle Radiologie, Universitätsklinikum des Saarlandes, Homburg, Deutschland
^8^ Rhein Main Vascular Center, Klinik für vaskuläre und endovaskuläre Chirurgie, Asklepios Kliniken Langen, Paulinen Wiesbaden und Seligenstadt
^9^ Abt. für Diagnostische und Interventionelle Radiologie, Universitätsklinikum Tübingen, Tübingen, Deutschland
^10^ Institut für Qualität und Transparenz im Gesundheitswesen (IQTIG), Berlin, Deutschland
^11^ Klinik für Gefäßchirurgie, Universitätsklinikum Ruppin-Brandenburg, Medizinische Hochschule Brandenburg, Neuruppin, Deutschland
^12^ Gefäßchirurgie, Alexianer St. Josefs Krankenhaus Potsdam, Potsdam, Deutschland
^13^ Sektion Pflegeforschung, Deutsche Interdisziplinäre Vereinigung für Intensiv- und Notfallmedizin e.V., Berlin, Deutschland
^14^ Deutscher Berufsverband für Pflegeberufe (DBfK) Bundesverband e.V., Berlin, Deutschland
^15^ Pflegedirektion, Alexianer St. Josefs Krankenhaus Potsdam, Potsdam, Deutschland
^16^ Klinik für Gefäßchirurgie, Immanuel Albertinen Diakonie, Hamburg, Deutschland
^17^ Klinik für Gefäßmedizin, Universitätsklinikum Hamburg-Eppendorf, Hamburg, Deutschland
^18^ Abteilung für Herz- und Gefäßchirurgie, Universitätsklinikum Freiburg, Freiburg, Deutschland
^19^ Medizinische Fakultät, Albert Ludwigs Universität Freiburg
^20^ Kompetenz-Centrum Qualitätssicherung (KCQ), Medizinischer Dienst Baden-Württemberg, Tübingen, Deutschland
^21^ Deutsches Herzzentrum der Charité (DHZC), Klinik für Herz-, Thorax- und Gefäßchirurgie, Berlin, Deutschland
^22^ Charité – Universitätsmedizin Berlin, Corporate Member of Freie Universität Berlin and Humboldt Universität zu Berlin, Berlin, Germany
^23^ Abteilung für Gefäß- und endovaskuläre Chirurgie, Asklepios Klinik Altona, Asklepios Medical School, Hamburg, Deutschland
^24^ Klinik für Anästhesiologie, Intensiv- und Schmerzmedizin, SANA Klinikum Offenbach, Offenbach, Deutschland
^25^ Klinik für Vaskuläre und Endovaskuläre Chirurgie, Uniklinik Münster, Münster, Deutschland
^26^ Abteilung für Gefäß- und Endovaskularchirurgie, Universitätsklinikum Frankfurt, Frankfurt, Deutschland
^27^ Interessenvertretung Patienten-&-Versicherte, Sörgenloch, Deutschland
^28^ Universitäres Gefäßzentrum Ostbayern, Abteilung für Gefäßchirurgie, Universitätsklinikum Regensburg, Regensburg, Deutschland
^29^ Uniklinikum Dresden, Bereich Gefäß- und Endovaskuläre Chirurgie Uniklinikum Dresden, Dresden, Deutschland
^30^ Deutsche Gesellschaft für Pflegewissenschaft e.V., Duisburg, Deutschland
^31^ Deutsche Gesellschaft für Chirurgie e.V., Berlin, Deutschland
^32^ Klinik für Gefäßchirurgie, Gefäßzentrum Barmherzige Brüder Regensburg, Regensburg, Deutschland
^33^ Katholische Akademie für Berufe im Gesundheits- und Sozialwesen, Regensburg, Deutschland
^34^ Intensivpflege, Asklepios Klinik Wandsbek, Hamburg, Deutschland
^35^ Deutsche Gesellschaft für Fachkrankenpflege und Funktionsdienste e.V., Berlin, Deutschland
^36^ St. Josefs Hospital Wiesbaden, Wiesbaden, Deutschland
^37^ BQS Institut, Hamburg, Deutschland
^38^ Klinikum Nürnberg und Paracelsus Medizinische Privatuniversität, Nürnberg
^39^ Deutsche Interdisziplinäre Vereinigung für Intensiv- und Notfallmedizin e.V., Berlin, Deutschland
^40^ Klinik Für Unfall-, Hand- und Wiederherstellungschirurgie, Universitätsklinikum Essen, Universität Duisburg-Essen, Deutschland
^41^ Abteilung für Anästhesiologie, Intensiv- und Notfallmedizin, Asklepios Klinik Wandsbek, Hamburg, Deutschland
^42^ Universitätsklinik Frankfurt, Klinik für Anästhesiologie, Intensivmedizin und Schmerztherapie, Frankfurt am Main, Deutschland
^43^ Klinik für Gefäßmedizin, Gefäßchirurgie und Endovaskuläre Chirurgie, Klinikum Darmstadt, Darmstadt, Deutschland

Die Einladung wurde über verschiedene Verteiler, Fachgesellschaften und Berufsverbände verteilt. Bei kurzfristigem Interesse an einer Mitwirkung: [behrendt@hamburg.de](mailto:behrendt@hamburg.de)

1. Behrendt, CA., Adili, F., Böckler, D. et al. Das Qualitätssicherungs- und Deviceregister des Deutschen Instituts für Gefäßmedizinische Gesundheitsforschung der DGG im Zeitalter von COVID-19, Big Data und künstlicher Intelligenz. Gefässchirurgie 27, 317–320 (2022). <https://doi.org/10.1007/s00772-022-00916-y> [↑](#footnote-ref-1)
2. <https://www.g-ba.de/downloads/62-492-3032/QBAA-RL_2022-12-07_iK-2023-01-01.pdf>, zugegriffen am 6. Juli 2023 [↑](#footnote-ref-2)
3. Waggoner J, Carline JD, Durning SJ. Is There a Consensus on Consensus Methodology? Descriptions and Recommendations for Future Consensus Research. Acad Med. 2016 May;91(5):663-8. doi: <https://doi.org/10.1097/ACM.0000000000001092> PMID: 26796090. [↑](#footnote-ref-3)
4. Behrendt CA, Thomalla G, Rimmele DL, Petersen EL, Twerenbold R, Debus ES, Kölbel T, Blankenberg S, Schmidt-Lauber C, Peters F, Zyriax BC. Editor's Choice - Prevalence of Peripheral Arterial Disease, Abdominal Aortic Aneurysm, and Risk Factors in the Hamburg City Health Study: A Cross Sectional Analysis. Eur J Vasc Endovasc Surg. 2023 Apr;65(4):590-598. doi: <https://doi.org/10.1016/j.ejvs.2023.01.002>. PMID: 36634745. [↑](#footnote-ref-4)
5. Powell JT, Wanhainen A. Analysis of the Differences Between the ESVS 2019 and NICE 2020 Guidelines for Abdominal Aortic Aneurysm. Eur J Vasc Endovasc Surg. 2020 Jul;60(1):7-15. doi: <https://doi.org/10.1016/j.ejvs.2020.04.038>. PMID: 32439141. [↑](#footnote-ref-5)
6. Behrendt, CA., Larena-Avellaneda, A., Kölbel, T. et al. Was Sie schon immer zur Behandlung des abdominalen Aortenaneurysmas in Deutschland wissen wollten: Real-World-Evidenz, Trends und offene Fragen. Gefässchirurgie 26, 252–260 (2021). [↑](#footnote-ref-6)
7. Mao J, Behrendt CA, Falster MO, Varcoe RL, Zheng X, Peters F, Beiles B, Schermerhorn ML, Jorm L, Beck AW, Sedrakyan A. Long-term Mortality and Reintervention after Endovascular and Open Abdominal Aortic Aneurysm Repairs in Australia, Germany, and the US. Ann Surg. 2022 Nov 28:10.1097/SLA.0000000000005768. doi: <https://doi.org/10.1097/SLA.0000000000005768>. PMID: 36538620; PMCID: PMC10225011. [↑](#footnote-ref-7)
8. Kühnl A, Erk A,Trenner M, Salvermoser M, Schmid V, Eckstein HH: Incidence, treatment and mortality in patients with abdominal aortic aneurysms—an analysis of hospital discharge data from 2005–2014. Dtsch Arztebl Int 2017; 114: 391–8. DOI: <https://doi.org/10.3238/arztebl.2017.0391> [↑](#footnote-ref-8)
9. Behrendt CA, Sedrakyan A, Rieß HC, Heidemann F, Kölbel T, Petersen J, Debus ES. Short-term and long-term results of endovascular and open repair of abdominal aortic aneurysms in Germany. J Vasc Surg. 2017 Dec;66(6):1704-1711.e3. doi: <https://doi.org/10.1016/j.jvs.2017.04.040>. PMID: 28780975. [↑](#footnote-ref-9)
10. Schmitz-Rixen, T., Löffler, AK., Steinbauer, M. et al. Versorgung des intakten abdominellen Aortenaneurysmas (AAA) 2020/2021. Gefässchirurgie 28, 131–139 (2023). <https://doi.org/10.1007/s00772-022-00967-1> [↑](#footnote-ref-10)
11. Boyle JR, Mao J, Beck AW, Venermo M, Sedrakyan A, Behrendt CA, Szeberin Z, Eldrup N, Schermerhorn M, Beiles B, Thomson I, Cassar K, Altreuther M, Debus S, Johal AS, Waton S, Scali ST, Cromwell DA, Mani K. Editor's Choice - Variation in Intact Abdominal Aortic Aneurysm Repair Outcomes by Country: Analysis of International Consortium of Vascular Registries 2010 - 2016. Eur J Vasc Endovasc Surg. 2021 Jul;62(1):16-24. doi: 10.1016/j.ejvs.2021.03.034. Epub 2021 Jun 16. PMID: 34144883. [↑](#footnote-ref-11)
12. Beck AW, Sedrakyan A, Mao J, Venermo M, Faizer R, Debus S, Behrendt CA, Scali S, Altreuther M, Schermerhorn M, Beiles B, Szeberin Z, Eldrup N, Danielsson G, Thomson I, Wigger P, Björck M, Cronenwett JL, Mani K; International Consortium of Vascular Registries. Variations in Abdominal Aortic Aneurysm Care: A Report From the International Consortium of Vascular Registries. Circulation. 2016 Dec 13;134(24):1948-1958. doi: 10.1161/CIRCULATIONAHA.116.024870. Epub 2016 Oct 26. PMID: 27784712; PMCID: PMC5147037. [↑](#footnote-ref-12)
13. <https://www.swiss-icu.ch/de/richtlinien.html>; zugegriffen am 25.07.2023 [↑](#footnote-ref-13)
14. <https://www.ficm.ac.uk/standardssafetyguidelinesstandards/guidelines-for-the-provision-of-intensive-care-services>; zugegriffen am 25.07.2023 [↑](#footnote-ref-14)
15. Empfehlung zur Struktur und Ausstattung von Intensivstationen 2022 (Erwachsene) Version vom 2.November 2022. Waydhas C., Riessen R., Markewitz A., Hoffmann F., Frey L., Böttiger B.W., Brenner S., Brenner T., Deffner T., Deininger M., Janssens U., Kluge S., Marx G., Schwab S., Unterberg A., Walcher F., van den Hooven T. [↑](#footnote-ref-15)
